# Further Characterization of the Capsule-Like Complex (CLC) Produced by *Francisella tularensis* Subspecies *tularensis*: Protective Efficacy and Similarity to Outer Membrane Vesicles

**DOI:** 10.3389/fcimb.2018.00182

**Published:** 2018-06-15

**Authors:** Anna E. Champion, Aloka B. Bandara, Nrusingh Mohapatra, Kelly M. Fulton, Susan M. Twine, Thomas J. Inzana

**Affiliations:** ^1^Department of Biomedical Sciences and Pathobiology, Virginia-Maryland College of Veterinary Medicine, Virginia Tech, Blacksburg, VA, United States; ^2^Institute for Biological Sciences, National Research Council Canada, Ottawa, ON, Canada; ^3^Virginia Tech Carilion School of Medicine, Roanoke, VA, United States

**Keywords:** *Francisella tularensis*, virulence factors, capsule-like complex, outer membrane vesicles, protection, immune response

## Abstract

*Francisella tularensis* is the etiologic agent of tularemia, and subspecies *tularensis* (type A) is the most virulent subspecies. The live vaccine strain (LVS) of subspecies *holarctica* produces a capsule-like complex (CLC) that consists of a large variety of glycoproteins. Expression of the CLC is greatly enhanced when the bacteria are subcultured in and grown on chemically defined medium. Deletion of two genes responsible for CLC glycosylation in LVS results in an attenuated mutant that is protective against respiratory tularemia in a mouse model. We sought to further characterize the CLC composition and to determine if a type A CLC glycosylation mutant would be attenuated in mice. The CLCs isolated from LVS extracted with 0.5% phenol or 1 M urea were similar, as determined by gel electrophoresis and Western blotting, but the CLC extracted with urea was more water-soluble. The CLC extracted with either 0.5% phenol or 1 M urea from type A strains was also similar to the CLC of LVS in antigenic properties, electrophoretic profile, and by transmission electron microscopy (TEM). The solubility of the CLC could be further enhanced by fractionation with Triton X-114 followed by N-Lauroylsarcosine detergents; the largest (>250 kDa) molecular size component appeared to be an aggregate of smaller components. Outer membrane vesicles/tubules (OMV/T) isolated by differential centrifugation and micro-filtration appeared similar to the CLC by TEM, and many of the proteins present in the OMV/T were also identified in soluble and insoluble fractions of the CLC. Further investigation is warranted to assess the relationship between OMV/T and the CLC. The CLC conjugated to keyhole limpet hemocyanin or flagellin was highly protective against high-dose LVS intradermal challenge and partially protective against intranasal challenge. A protective response was associated with a significant rise in cytokines IL-12, IL-10, and IFN-γ. However, a type A CLC glycosylation mutant remained virulent in BALB/c mice, and immunization with the CLC did not protect mice against high dose respiratory challenge with type A strain SCHU S4.

## Introduction

*Francisella tularensis*, the etiologic agent of tularemia, is a Gram-negative coccobacillus capable of causing severe disease in animals and humans (Olsufjev, 1966). Infection can occur through several routes, which include the lungs (inhalation), ingestion of contaminated water or food (gastrointestinal), or a break in the skin and mucous membranes (ulcero-gandular) (Keim et al., 2007; Sjöstedt, 2007). *F. tularensis* is classified as a Tier I select agent by the U.S. Center for Disease Control due to its ease of dispersal, persistence in the environment, and its high infectivity and potential lethality (Dennis et al., 2001). *F. tularensis* subspecies *tularensis* (type A) is the most virulent biotype and found predominately in North America. Subspecies *tularensis* is capable of causing lethal disease in up to 30% of untreated cases with as few as 10 organisms (via inhalation) (McCrumb, 1961; Brooks and Buchanan, 1970). *F. tularensis* subspecies *holarctica* (type B), is less virulent for humans, but is responsible for most tularemia outbreaks in Europe and can cause death if untreated (Chen et al., 2003; Kantardjiev et al., 2006). Research over the last decade has expanded our understanding of the pathogenesis of this facultative intracellular pathogen and progress has been made on development of a vaccine (Jones et al., 2014; Sunagar et al., 2016). Currently there is no approved, licensed vaccine for tularemia. However, a live attenuated strain of subspecies *holarctica* was developed by researchers in the Soviet Union, and modified by U.S. investigators. This attenuated strain, later referred to as the live vaccine strain (LVS), conferred protection against aerosol challenge with virulent type A strains (Saslaw et al., 1961). Although LVS induces improved protection compared to killed strains, it is still not adequately protective against high dose respiratory exposure. Due to the need for improved protection, the potential for virulence in some immune-compromised individuals, and concern over the strain's stability (phase variable gray variants readily develop and are not protective), LVS is not approved for use as a vaccine to the public. Given the intracellular nature of *F. tularensis* and the success of LVS as a vaccine, a live attenuated type A strain with a defined mutation would logically be expected to be the most efficacious to induce protective immunity against infection. Targeted mutations that have been successful in attenuating *F. tularensis* have included surface components such as the lipopolysaccharide (LPS) and some outer membrane proteins (OMPs) (Thomas et al., 2007; Meibom et al., 2009; Qin et al., 2009; Reed et al., 2014), and the *Francisella* pathogenicity island (FPI) (Ozanic et al., 2016).

Investigation of the surface components of *F. tularensis* has markedly increased over the past 10–15 years. Some of the known surface components include LPS, O-antigen capsule, pili, several outer membrane proteins, outer membrane vesicles and tubes (OMV/T), and the capsule-like complex (CLC). The LPS has been the most thoroughly studied surface antigen, and its role in virulence and resistance to complement-mediated bactericidal activity is well-documented (Hartley et al., 2004; Li et al., 2007; Raynaud et al., 2007; Sebastian et al., 2007). Deletion of the O-antigen in types A and B strains results in a highly attenuated mutant that confers protection against challenge with type B strains, but not type A infection (Li et al., 2007; Raynaud et al., 2007; Sebastian et al., 2007; Thomas et al., 2007). In addition, a capsule comprised of O-antigen sugars is present in types A and B strains, and is necessary for intracellular growth (Apicella et al., 2010). While an O-antigen capsule-deficient mutant is attenuated, this mutant also does not provide adequate protection against type A *F. tularensis* (Apicella et al., 2010; Lindemann et al., 2011). OMV, common to Gram-negative bacteria, are also produced by *Francisella* spp., but the majority of that work has been described in *F. novicida* (Pierson et al., 2011; McCaig et al., 2013), a more distant subspecies of *F. tularensis*. Several proteins recognized as virulence factors, such as FopB, TolC, GroEL, as well as the FPI proteins IglABC and PdpAB, are also present in the OMV (Nano et al., 2004; Oyston, 2008; Pierson et al., 2011). Furthermore, *F. novicida* OMV have successfully been used as a vaccine against challenge with *F. novicida*, but that does not indicate that OMVs would protect against the more virulent *F. tularensis* type A and B strains (Pierson et al., 2011). However, Huntley et al. (2008) have shown that outer membranes consisting of native OMPs provide limited protection of mice against challenge with 40 colony forming units (CFU) of type A strain SCHU S4. Golovliov *et al*. reported that a capsular/vesicular material surrounds phagocytized *F. tularensis* and that this material could be shed during infection as a way of masking the bacteria from the host (Golovliov et al., 2003).

Another novel surface component that has been identified in *F. tularensis* is the CLC (Bandara et al., 2011). The CLC appears as an electron dense material around the cell surface when visualized by transmission electron microscopy (TEM), and its presence requires subculture under specific growth conditions and components (Cherwonogrodzky et al., 1994). After semi-purification, the CLC was found to consist of about 10% carbohydrate that is predominately glucose, galactose, and mannose (Bandara et al., 2011). However, the CLC appears to contain many proteinase-resistant proteins, glycoproteins, and possibly a high molecular size (HMS) carbohydrate/glycoprotein that presents as multiple bands or a diffuse banding between 150 and 250-kDa (Bandara et al., 2011). Identification of novel glycoproteins, or the presence of previously identified *F. tularensis* glycoproteins, such as DsbA or the Pil proteins, in the CLC has not yet been established. The production of HMS carbohydrate was also found to be dependent on growth in medium that mimicked growth factors present in the host (Brain Heart Infusion, BBL) (Zarrella et al., 2011). A definitive link between the HMS carbohydrate, described by Zarrella et al., and the CLC has yet to be established (Zarrella et al., 2011). The locus responsible for glycosylation of some proteins (FTT0789-FTT0800), such as DsbA, in *F. tularensis* type A and B strains is also responsible for glycosylation of the CLC (Larsson et al., 2005; Bandara et al., 2011) (Balonova et al., 2010; Thomas et al., 2011). We previously demonstrated that when two glycosyl transferases (FTT0798-99) in this locus were deleted in LVS (Bandara et al., 2011) or *F. novicida* (Freudenberger Catanzaro et al., 2017), CLC production was significantly diminished and both species were highly attenuated. In LVS, and to a lesser extent *F. novicida*, the CLC-deficient strains were protective against challenge with the parent strain.

Further identification of the many individual components of the CLC could result in a better understanding of the CLC's role in virulence and help elucidate potential genetic targets that could be useful for vaccine development. In this work we further characterized the *F. tularensis* CLC from both type A and LVS strains through enhancing solubility, further compositional analysis, and comparison to OMV/T. Immunization of mice with the CLC conjugated to protein stimulated a selective cytokine response and was protective against LVS challenge. However, a CLC glycosylation mutant of type A *F. tularensis* remained virulent in mice, and immunization of mice with the CLC did not provide adequate protection against respiratory challenge with SCHU S4. Further analysis of the CLC and its components could prove advantageous in elucidating the role of CLC in pathogenesis.

## Materials and methods

### Bacterial strains and growth conditions

The bacterial strains used and their sources are listed in Table 1. LVS mutants lacking LPS O-antigen were used for CLC isolation and characterization to eliminate contamination of CLC extracts with O-antigen sugars. *F. tularensis* strains were cultured from frozen stock suspensions onto brain heart infusion agar (BHIA) (Becton-Dickinson, Franklin Lakes, NJ) supplemented with 0.1% L-cysteine hydrochloride monohydrate (Sigma-Aldrich, St. Louis, MO) (BHIA), Chamberlain's defined medium (CDM) (Cherwonogrodzky et al., 1994) with 1.5% glucose, or Modified Mueller Hinton (MH) medium supplemented with 0.1% glucose, 0.025% ferric pyrophosphate, and 2% IsoVitaleX (Becton-Dickinson), and incubated at 37°C in 6% CO_2_, unless otherwise stated. Supplemented BHI medium was the standard medium used to routinely culture *F. tularensis*. Supplemented CDM medium was used to enhance CLC production, as described (Bandara et al., 2011). Supplemented MH medium was used based on evidence that *F. tularensis* grown in MHB medium lacks the cell surface HMS carbohydrates/glycoproteins that are expressed by bacteria grown in BHI medium and during growth in mammalian hosts (Zarrella et al., 2011). For culture in broth, *F. tularensis* strains were grown with shaking (175 rpm) in BHI broth (BHIB) at 37°C, or CDM broth (CDMB) at 32°C. For selection of recombinant strains 15 μg kanamycin (Kan)/ml was added to the stated medium. For enhancement of surface CLC, the bacteria were subcultured in CDMB daily for 10 consecutive passages and indicated by the extension “P10” on the strain name. For CLC preparation, CDMB-subcultured *F. tularensis* was grown on CDM agar (CDMA) in 150-mm × 15-mm petri dishes, and incubated at 32°C in 6% CO_2_ for 5 days. All experiments with LVS and mutants were carried out in biosafety level (BSL)-2 facilities in an approved biosafety cabinet. All experiments with type A strains TI0902 and SCHU S4 were carried out in a biosafety level-3 (BSL-3) facility in an approved biosafety cabinet in the college's infectious disease unit (IDU). All investigators working with select agents have FBI and CDC clearance and approval. The current CDC approval number for the BSL3/ABSL3 facility of the IDU is C20111027-1280.

**Table 1:** *Francisella tularensis* bacterial strains used in this study.

**Strain**	**LPS/O-antigen**	**O-antigen capsule**	**CLC**	**Attenuated**	**Source**
LVS	+	+	+	N	May Chu, CDC
LVS_P10^a^	+	+	+++	N	Bandara et al., 2011
LVSΔ1423-22_P10^a^	+	+	−	Y	Bandara et al., 2011
WbtI_G191V_	−	−	+	Y	Li et al., 2007
WbtI_G191V__P10^a^	−	−	+++	Y	Bandara et al., 2011
WbtI_G191V_Δ1423-22_P10^a^	−	−	−	Y	Bandara et al., 2011
SCHU S4_P10^a^	+	+	+++	N	Mark Wolcott, USAMRIID
SCHU S4Δ0798/0799_P10^a^	+	+	−	N	This work
TI0902	+	+	+	N	Inzana et al., 2004
TI0902_P10^a^	+	+	+++	N	This work
TIGB03	−	+	+	Y	Modise et al., 2012
TIGB03[*wbtk*+]	+	+	+	N	This work

a *The suffix P10 indicates the strain has been subcultured daily for 10 days in Chamberlain's defined medium (CDM) broth followed by culture on CDM agar at 32°C for several days to enhance expression of CLC*.

### Mutagenesis of the Type A CLC glycosylation locus

The suicide vector pJC84 (generously provided by Jean Celli, Washington State University, Pullman, WA) was used to generate an in-frame deletion of FTT_0798 and 0799 genes in the *F. tularensis* strain SCHU S4. The deletion construct of these genes was carried out as described (Mohapatra et al., 2013). In brief, PCR amplified products of about 1100 bp of an upstream region (forward primer—ACG CGT CGA CGA AGT ATT TAA AAG GAT ATT TTC ACG TAG and reverse primer—CGC GGA TCC ATT TAC CTT AAG AGT ATT AAT CTT TAA ATA AGA AG) and downstream region (forward primer—CGC GGA TCC AAA ATT TTA AGG AAT GAA ATG AAA ACC T and reverse primer—TCC CCC CGG GCT TTC TGT GCA AAT ATT TAC AAA GG) of FTT_0798 and 0799 was cloned into the pJC84 plasmid using suitable restriction sites. All plasmid constructs were verified by sequencing, and glycerol stocks were frozen at −80°C for further use. The deletion constructs were transformed into SCHU S4 by electroporation as described (McRae et al., 2010). Kanamycin-resistant transformants were tested for integration of the allelic replacement plasmid using suitable primer combinations (forward primer—CTA GCT AGC AGG AGA CAT GAA CGA TGA ACA TC, reverse primer—GGG ACG TCG GAT TCA CCT TTA TGT TGA TAA G and forward primer—ATC AGC TCA CTC AAA GGC GG and reverse primer—GGG ACG TCG ATT AAG CAT TGG TAA CTG TCA GAC C). The positive clones were subjected to sucrose counter selection as described (McRae et al., 2010). Sucrose-resistant clones were patched on MH-kanamycin agar to verify loss of the kanamycin-resistance marker, and colony PCR was performed to detect clones with allelic replacement within the correct chromosomal locus using FTT_0798 and 0799 gene specific primers, which are identical to primers used for detection of FTL_1422 and FTL_1423 from LVS (Bandara et al., 2011). Mutagenesis of the glycosylation locus in one clone (SCHU S4Δ0798-99) was confirmed by the loss of carbohydrate content using the anthrone assay (Scott and Melvin, 1953) as previously described (Freudenberger Catanzaro et al., 2017).

### CLC extraction

To avoid the presence of O-antigen sugars in CLC extracts, LVS O-antigen mutant WbtI_G191V__P10 (Li et al., 2007) was used for most CLC extractions. To enhance for CLC expression, the bacteria were subcultured daily for 10 days in CDMB at 37°C with shaking, and then grown on CDMA for 5 days with 6% CO_2_ at 32°C, as previously described (Bandara et al., 2011). For some experiments, the cells were also grown on BHI or MH agar. To determine if solubility of the CLC could be improved, the bacteria were extracted with a variety of chaotropic agents. The bacteria were scraped off the plates, and gently washed twice with 10 mM HEPES (4-2-*hydroxyethyl*)-1-piperazineethanesulfonic acid) before extraction of the CLC. The bacteria were suspended to 2 × 10^10^ (CFU)/ml, determined spectrophotometrically, pelleted by centrifugation, and suspended in 1 ml of various chaotropic agents (e.g., low/high pH, urea, phenol, guanidine, neutral buffers) for 15 min at room temperature to determine the extraction buffer that maximized solubility of the CLC (Supplementary Table 1). The bacteria were removed by centrifugation and acidic samples were adjusted to pH 7 with 5N NaOH to avoid protein degradation. Supernatants were either used directly or excess ethanol was added to precipitate large molecular size components. Urea (1 M) was determined to be the most effective extraction buffer, and therefore all CLC extractions described in this study utilized 1 M urea or 0.5% phenol, as described previously (Bandara et al., 2011). Briefly, the bacteria were scraped off two agar plates with 20 mls of 1 M urea or 0.5% phenol. The suspended cells were incubated at room temperature for 15 min, harvested by centrifugation (10,000 × g for 15 min), and the supernatant retained. The wet weight of the cell pellets was determined, and the CLC volumes adjusted based on the mass between parent and mutant strains to normalize the extracts. For type A strains extractions were carried out in a BLS-3 facility and complete loss of cell viability was verified before removal of any material from the BSL-3 laboratory. After urea-extracted supernatants were harvested, sodium acetate was added to a final concentration of 30 mM and the CLC was precipitated with 5 volumes of cold 95% ethanol. A portion of this crude supernatant was concentrated 5- to 10-fold by ultrafiltration through a 100-kDa Centriprep filter (Millipore, Darmstadt, Germany).

For some experiments, WbtI_G191V__P10 was grown in CDM, BHI, or MH broth (100 ml) with shaking at 180 rpm at 32 or 37°C. The broth cultures were grown to 10^9^ CFU/ml, determined spectrophotometrically and confirmed by viable plate count. The cells were removed by centrifugation (10,000 × g for 15 min), and 5 volumes of 95% ethanol were added to the supernatant and held at −20°C overnight. The resulting precipitate was harvested and resuspended in 5–10 ml of sterile water and lyophilized. The cell pellet was resuspended in 20 ml of 1 M urea and extracted for CLC, as described above.

### Electrophoretic analysis of CLC

Ten μl of CLC extract was analyzed by sodium dodecyl sulfate-polyacrylamide gel electrophoresis (SDS-PAGE) on either 4–8% NuPAGE or 4–12% NuPAGE gels (Invitrogen, Grand Island, NY). The gels were stained with either the SilverSnap kit (Pierce, Rockford, IL) or by StainsAll/silver stain (Sigma), as described (Bandara et al., 2011). Carbohydrate in the gels developed with StainsAll/silver appeared as blue bands, proteins were pink, lipids yellow, and glycoproteins appeared purple. Unmodified, ionic proteins are not stained and appear white, or are negatively stained by StainsAll/silver stain. To avoid interference by LPS O-antigen, only extracts from O-antigen mutants were examined by gel electrophoresis. As a control, excess 95% cold ethanol was added to media without bacterial growth and the precipitate was solubilized and analyzed as described above.

For fractionation analysis, 100 μg (lyophilized weight) of urea-extracted CLC from O-antigen mutant WbtI_G191V__P10 was electrophoresed through the Gel Elution Liquid Fraction Entrapment Electrophoresis (GELFREE) 8100 (Expedeon, San Diego, CA) protein fractionation system. This system uses cartridges to fractionate proteins over a mass range of 3.5–500 kDa into user-selectable, liquid phase molecular weight fractions. Urea-extracted CLC was desalted by Zeba spin desalting columns (7K MWCO) (Pierce) prior to fractionation and lyophilized. The desalted CLC (100 μg) was resuspended in 112 μl sterile distilled water, and 8 μl of 1 M DTT (dithiothreitol) and 30 μl of provided sample buffer was added. The sample was heated at 50°C for 10 min, cooled to room temperature, and fractionated into 12 fractions following the manufactures' instructions for the high molecular size 5% tris-acetate cartridge. Each fraction was then analyzed by SDS-PAGE.

To resolve insolubility issues that commonly occurred following concentration of even the urea-extracted CLC, purified CLC from WbtI_G191V__P10 and WbtI_G191V_Δ1423-22_P10 was fractionated using the detergents Triton X-114 and *N*-Lauroylsarcosine (sarkosyl) (Bordier, 1981). Samples from O-antigen and O-antigen/CLC mutants were normalized to equivalent amounts of wet weight because normalization based on protein content would be inaccurate due to substantial differences in glycosylation of CLC between the different mutants. Due to incubation on plates for several days, a viable plate count for bacterial cell numbers would also be inaccurate. Equal amounts (5 mg) of lyophilized, urea-extracted CLC from the O-antigen mutant and the CLC-deficient mutants were suspended in 5 ml of distilled water and Triton X-114 (Sigma) was added to a final concentration of 5% (v/v). The samples were vortexed for 1 min, maintained on ice at 4°C overnight, the samples were incubated at 37°C for 2 h, and then centrifuged at 3000 × g at room temperature for 10 min to separate the aqueous (TxS-A) and detergent phases (TxS-D). Both phases were removed carefully, dialyzed against distilled water, and lyophilized. The Triton X-114 insoluble pellet was resuspended in 1 ml of 1% sarkosyl, incubated at room temperature for 15 min, and the samples centrifuged at 5000 × g for 10 min at room temperature. The soluble supernatant was removed, and the insoluble pellet resuspended in 1 ml of distilled water, constituting the sarkosyl-soluble (TxI-SS) and sarkosyl-insoluble (TxI-SI) fractions, respectively. All fractions were analyzed on a 4–12% bis-tris NuPAGE gel (Invitrogen) and visualized by the SilverSnap (Pierce) silver stain kit.

### OMV extraction

OMV were purified from *F. tularensis* strains by one of two procedures. The method described for isolation of OMV and outer membrane tubules (OMT) from *F. novicida* (McCaig et al., 2013) was slightly modified. Briefly, bacterial cultures were grown in 1 L of CDMB for ~5 h with appropriate antibiotic to exponential phase, the bacteria removed by successive, differential low-speed centrifugation (5,000 × g, followed by 7500 × g, for 30 min each), and the supernatant passed through a 0.45 μm syringe filter (Nalgene, Rochester, NY). The filtered supernatant was concentrated from 1 L to about 50 ml using an Amicon tangential flow filtration unit with a 100-kDa molecular mass cutoff membrane (Millipore, Darmstadt, Germany). The concentrated cell-free medium was then ultracentrifuged at100,000 × g for 1 h at 4°C. The OMV/T pellet was resuspended in 10 mM HEPES (pH 7.5) containing 0.05% sodium azide, and ultracentrifuged again. The pelleted OMV/T was resuspended in 1–2 ml of HEPES buffer with sodium azide and stored at −20°C until use.

The second OMV method (referred to as OMV_A_) used more closely mimics the CLC extraction procedure, and was modified from a previously published method for *Brucella* (Gamazo et al., 1989; Avila-Calderón et al., 2012). Overnight cultures of *F. tularensis* strains from CDMB were grown at 32°C on 5 CDMA plates with appropriate antibiotic. Bacteria were scraped off the agar plates and suspended in 100 ml sterile phosphate buffered saline (PBS), pH 7.3. The bacterial cells were removed by centrifugation at 10,000 × g for 30 min. The supernatant was passed through a 0.45 μm filter (Millipore) and sodium azide was added to 0.05%, final concentration. The supernatant was ultracentrifuged at 100,000 × g for 2 h at 4°C. The OMV pellet was washed twice with PBS and the OMV suspended in 1 ml of sterile PBS. The OMV_A_ were aliquoted and stored at −20°C. Total protein concentrations of OMV from each method were determined using the BCA protein kit (Pierce), as per manufacturer's instructions.

### CLC compositional analysis

Urea-extracted CLC was concentrated using a 100-kDa Centriprep filter unit (Millipore), and the sample separated into soluble and insoluble fractions (material that precipitates following concentration), as described above. Trypsin digests of the fractions were analyzed by nano-liquid chromatography-mass spectrometry (nLC-MS/MS using a “CapLC” (capillary chromatography system) (Waters) coupled to a “QTOF Ultima” hybrid quadrupole time-of-flight mass spectrometer (Waters) as described (Fulton et al., 2015). Peptide extracts were injected into a 75-μm internal diameter × 150-mm PepMap C_18_ nanocolumn (Dionex/LC packings), and resolved by gradient elution (5–75% acetonitrile, 0.l2% formic acid in 30 min, 350 nL/min). MS/MS spectra were acquired on doubly, triply, and quadruply charged ions. Peak lists were automatically generated by ProteinLynx (Waters) with the following parameters: smoothing—four channels, two smoothes, Savitzky Golay mode; centroid—minimum peak width at half height of four channels, centroid top 80%. nLC–MS/MS spectra of the tryptic peptides were searched against the NCBInr (NCBInr 20050724; 2693904 sequences) and *F. tularensis* LVS genome sequence using MASCOT 2.0.1 (Matrix Science, UK) to identify protein homologs. The following parameters were used for mass spectral identification: peptide tolerance of 1.5 Da, MS/MS tolerance of 0.8 Da, possible one missed cleavage site, variable modifications including carbamidomethylation of cysteine residues, oxidation of methionine, formation of pyroglutamate at *N*-terminal glycine, and *N*-terminal acetylation. Peptide identifications were accepted if they met all of the following criteria: MASCOT peptide score > 25, mass accuracy < 100 ppm. In addition, all MS/MS spectra were manually assessed for data quality and high confidence identification, requiring a clear series of high mass y ions and correct charge state assignment for all fragments. However, proteins that were heavily glycosylated could not be identified using this method.

Peptide composition of in-gel proteolytic digests were performed using trypsin (Promega, Madison, WI, USA) at a ratio of 30:1 (protein/enzyme, w/w) in 50 mM ammonium bicarbonate at 37°C overnight. The resulting protein digests were analyzed by nLC-MS/MSano-liquid chromatography as described above. In addition, ion pairing normal-phase liquid chromatography (IP-NPLC) was used for enrichment and identification of putative glycopeptides from the urea-extracted CLC of WbtI_G191V__P10, as described previously (Thomas et al., 2011).

Glycose composition of type A CLC was determined by combined GC/MS of the per-O-trimethylsilyl (TMS) derivatives of the monosaccharide methyl glycosides produced from the sample by acidic methanolysis, as described (Merkle and Poppe, 1994). Amino acid analysis of the urea-extracted CLC was performed by the University of California at Davis Proteomics Institute (Ozols, 1990; Kataoka et al., 2001).

### Transmission electron microscopy

*Francisella tularensis* strains were grown on CDMA (with appropriate antibiotic) for 5 days at 32°C. The cells were gently scraped into 0.1 M sodium cacodylate buffer (pH 7.5) containing 3% glutaraldehyde and turned end-over-end for 2 h. Type A strains were left in the fixative at 4°C for up to 5 days. Periodically, cell death was verified by streaking 50 μl aliquots of fixed cells onto CDMA and incubating for 5 days. If no growth occurred after this time period the cells were removed from the BSL-3 facility. Samples were bound to formvar-coated slot grids, stained with 0.5% uranyl acetate for 10 s, and viewed with a JEOL 100 CX-II transmission electron microscope (Ward and Inzana, 1994).

### Generation of hyperimmune rabbit serum

Antiserum to WbtI_G191V__P10 CLC was raised in a New Zealand white rabbit by subcutaneous immunization of 100 μg phenol-extracted purified CLC mixed 1:1 in Freund's complete adjuvant, injected into 6 sites. Two weeks later, the immunization was repeated with 100 μg phenol-extracted purified CLC in Freund's incomplete adjuvant. Two weeks after this injection, the rabbit was immunized intravenously with 500 μg CLC at weekly intervals until hyperimmune serum (ELISA titer > 1:6,400) was obtained.

### CLC-protein conjugation and mouse challenge

CLC was purified from LVS O-antigen mutant WbtI_G191V__P10, which was enhanced for CLC production as previously described (Bandara et al., 2011). The purified CLC was conjugated to keyhole limpet hemocyanin (KLH) or purified *Salmonella* flagellin protein through an adipic acid dihydrazide (ADH) spacer as described (Schneerson et al., 1980). Briefly, the immunogenic protein carrier was conjugated to ADH at pH 4.7 with 1-ethyl-3-(3-dimethylaminopropy)carbodiimide (EDAC). Reactive groups on the glycose moiety of the CLC were generated with cyanogen bromide at pH 10.5, and conjugated to either KLH or flagellin through ADH at pH 8.5. The CLC-conjugate was eluted through a Sepharose S-300 size exclusion column and collected in the void volume. Fractions were tested for protein (absorbance at 280 nm), pooled, dialyzed against distilled water, and lyophilized. After lyophilization, if some insolubility was observed the samples were sonicated to regain clarity. To confirm conjugation was successful, CLC conjugates were analyzed by SDS-PAGE on 4–12% bis-tris gels with unconjugated CLC and the protein carrier as a control.

The CLC's protective efficacy against LVS challenge was assessed in groups of 6 BALB/C mice immunized intradermally (ID) with CLC-KLH or CLC-flagellin protein conjugates twice 6 weeks apart. The first and second immunizations were administered with Freund's Complete and Incomplete adjuvants, respectively. Mice were challenged with 5 × LD_50_ of LVS intranasally (IN) (5000 CFU) or ID with 2 × 10^7^ CFU. Blood samples were collected from mice prior to immunization, post-vaccination (at 6 weeks), and 3 days post-challenge. Serum samples were cryo-preserved at −80°C. Serum cytokine levels were determined in triplicate using the Bio-Plex ProTM Mouse Cytokine Th1/Th2 Assay (Bio-Rad, Hercules, CA) following the manufacturer's instructions.

To determine the protective efficacy of type A CLC to type A lethal challenge, groups of 4–8 mice were immunized subcutaneously (SC) with either crude CLC, a concentrated, semi-purified HMS CLC extract (both from SCHU S4 using 1 M urea), or water only. Mice were immunized with 50 μg of CLC ID twice 6 weeks apart or 3 months apart, with the first and second immunizations containing Freund's complete and Freund's incomplete adjuvant, respectively, or monophosphoryl lipid A (MPLA). Mice were IN-challenged with 100 or 150 CFU of *F. tularensis* SCHU S4 grown in supplemented BHI broth, washed in PBS, and adjusted to 10^9^ CFU/ml, determined spectrophotometrically and confirmed by viable plate count.

To assess if mutagenesis of the glycosylation locus attenuated type A strains as it does LVS and *F. novicida*, 6 BALB/c mice were each challenged IN with 100 CFU of SCHU S4 or SCHU S4Δ0798-99. Challenge doses were confirmed by viable plate count. Clinical symptoms of animals were recorded and any mice that became moribund were humanely euthanized with excess CO_2_.

### Statistics

Statistical analyses were performed using Microsoft Excel software (Redmond, WA) and GraphPad InStat software (La Jolla, CA). Protection data were analyzed by Fisher's Exact Test with Yates continuity correction. Differences in cytokine responses were measured by Tukey-Kramer multiple comparison test. Other variables were determined using students *t*-test and expressed as the mean ± standard deviation. Results with a *p* < 0.05 were considered statistically significant.

## Results

### Comparison of urea- and phenol-extracted CLC

**Figure 1:**
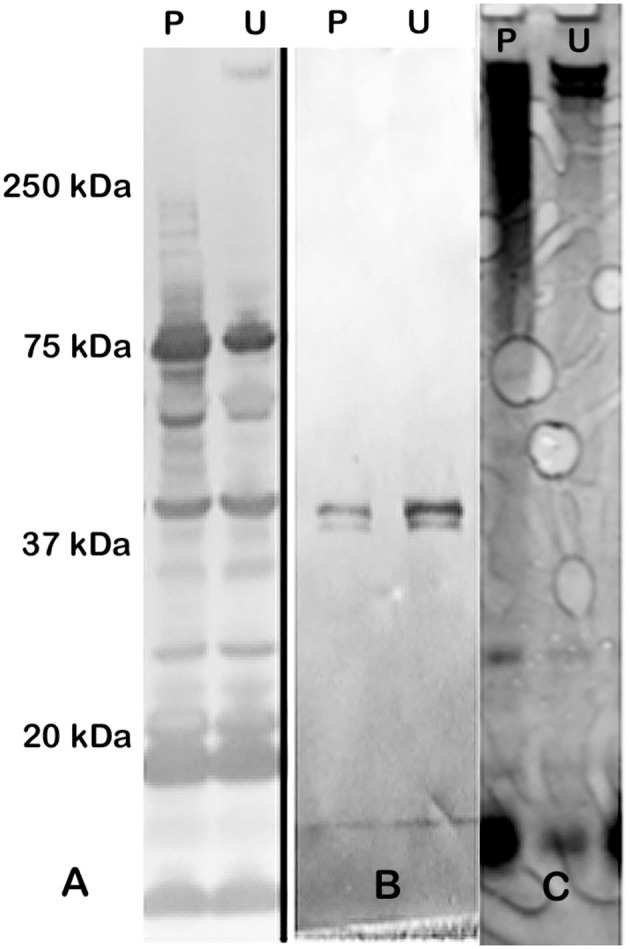
Western blot and SDS-PAGE silver stain of CLC. **(A)** Hyperimmune (HI) rabbit serum to CLC extracted with phenol and reacted to CLC extracted with phenol (P) or urea (U); **(B)** HI rabbit serum to phenol-extracted CLC adsorbed with CLC-deficient strain WbtI_G191V_Δ1423-22_P10 and reacted with phenol (P)—or urea (U)-extracted CLC. **(C)** Silver stain of CLC extracted with 0.5% phenol (P) or 1 M urea (U). Similar proteins were evident in both the phenol- and the urea-extracted CLC, but a HMS band was more prominent in the urea-extracted CLC. After the CLC HI sera was adsorbed with the CLC-deficient mutant only one band/doublet was evident at about 45-kDa, which is the same size as that fractionated with the GelFree 8100 (Figure 2).

Extraction of the CLC with 1 M urea greatly increased its solubility compared to extraction with 0.5% phenol (data not shown). However, when the urea was removed by dialysis with distilled water, or the material was concentrated through a 100-kDa filter > 2-fold, the material aggregated and became insoluble. The CLC extracted by urea and phenol were similar based on electrophoretic profile (Figure 1C) and by Western blotting with hyperimmune rabbit serum to CLC extracted with phenol (Figures 1A,B). Hyperimmune serum to CLC extracted with urea resulted in similar Western blot results (not shown). When immune serum was adsorbed with mutant WbtI_G191V_Δ1423-22_P10, most of the proteins were eliminated, except for a doublet at about 45-kDa (Figure 1B). The high molecular size band/smear at about 250-kDa was present in CLC extracted with phenol (P) or urea (U), but was more of a diffuse band in the phenol extract, possibly due to some degradation of proteins (Figure 1C). The band/smear at about 250-kDa was not present in the Western blot, likely because the HMS material did not transfer out of the gel as readily as lower molecular size material. Overall, electrophoretic, and immunological analyses indicated the preparations extracted with urea or phenol were very similar. Therefore, most of the extractions of CLC from *F. tularensis* were performed using 1 M urea.

**Figure 2:**
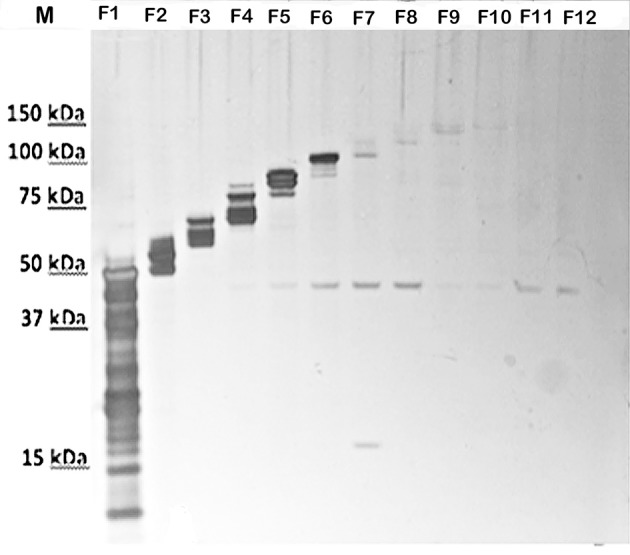
SDS-PAGE of silver-stained fractions of urea-extracted CLC recovered by the GelFree 8100 Fractionation System. F1-F12, fractions 1-12. Before fractionation staining of the CLC was evident as a smear above 150-kDa (shown in Figure 3), but after fractionation, the HMS material was absent and a 45-kDa band was present in fractions 5–8 and 11–12.

To attempt to further resolve the specific bands/components of the HMS material at about 250-kDa, the GelFree 8100 fractionation system, which separates analytes based on electrophoretic mobility, was used. Figure 2 shows the urea-extracted CLC of fractions F1-F12 following SDS-PAGE. Interestingly, there were no bands or smear above 150-kDa after fractionation. The larger protein bands last appeared faintly in fraction 9; a smaller 45-kDa band was clearly present in fractions 5–8 and 11–12. This 45-kDa band was also the only band evident on a Western blot of the CLC using immune serum absorbed with the CLC-deficient mutant (Figure 1B). Unfortunately, protein identification using mass spectrometry failed to identify this 45-kDa protein, likely due to glycosylation, which can suppress the MS signal from peptide fragment ions. Therefore, the HMS appeared to be an aggregate of (glyco)proteins, rather than a single protein or polysaccharide.

### Detergent fractionation

**Figure 3:**
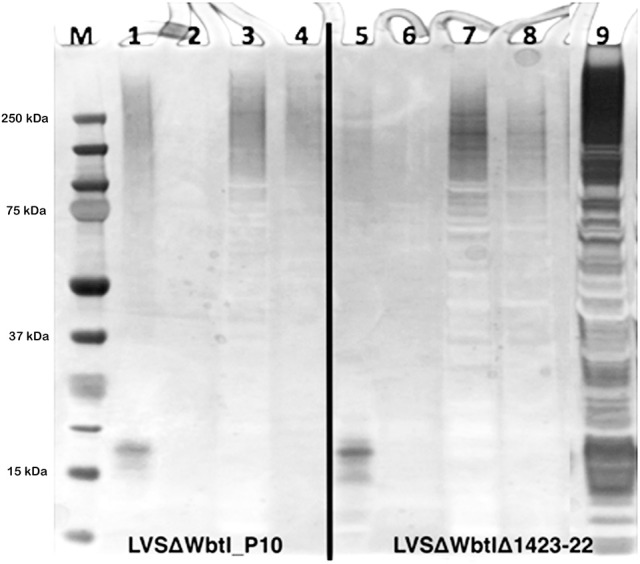
CLC fractionation using Triton X-114 and sarkosyl detergents. 4–12% NuPAGE SDS-PAGE stained using StainsAll/silver. Lanes: 1 and 5, TxS-A; 2 and 6, TxS-D; 3 and 7, TxI-SS; 4 and 8, TxI-SI; 9,) non-fractionated 1 M urea extract of CLC. CLC from WbtI_G191V__P10 fractioned predominantly into the TxS-A phase and the TxI-SS phase.

To further enhance the solubility of the CLC and clarify its composition, the CLC extracts were subjected to differential detergent stabilization using Triton X-114 alone or in combination with sarkosyl. Similar amounts of soluble protein were recovered in the Triton X-114 aqueous (TxS-A) and detergent (TxS-D) fractions of CLC from both the LVS O-antigen mutant and the double LVS O-antigen/CLC mutant (Table 2). O-antigen mutants were used to avoid interference of the O-antigen glycoses with carbohydrate components on glycoproteins of the CLC, particularly for analysis by SDS-PAGE and StainsAll/silver stain, which stains carbohydrate components (see below). However, most of the HMS diffuse material partitioned into the TxS-A phase. The Triton X-114 insoluble (TxI) material (recovered following Triton X-114 extraction and centrifugation) from CLC of the O-antigen mutant WbtI_G191V__P10 was far more soluble (~15:1) when subsequently extracted with 1% sarkosyl (TxI-SS) than the Triton X-114 insoluble material from the CLC of the O-antigen/CLC double glycosylation mutant WbtI_G191V_Δ1423-22_P10 (~2:1). When these fractions were analyzed by SDS-PAGE using StainsAll/Silver, substantial amounts of (glyco)protein in the Triton X-114-soluble aqueous phases (Figure 3, lanes 1 and 5), and soluble detergent phases (Figure 3, lanes 2 and 6) were not evident in the CLC preparations of WbtI_G191V__P10 or WbtI_G191V_Δ1423-22_P10, except proteins(s) at about 18-kDa from the TxS-A fractions. The increased amount of proteinaceous material solubilized by sarkosyl and visualized with StainsAll/Silver (Figure 3, lanes 3 and 7) was substantial (StainsAll negatively stains proteins, which appear as ghost bands on the gel), and much less protein remained in the sarkosyl-insoluble fraction (pellet recovered following sarkosyl extraction and centrifugation) (Figure 3, lanes 4 and 8; lane 9 contains unfractionated CLC). Overall, a greater amount of protein was present in the CLC of WbtI_G191V_Δ1423-22_P10 compared to the O-antigen mutant. Although the glycose-deficient strain made much less CLC than WbtI_G191V__P10, when compared 1:1 by weight, the CLC extracted from the CLC glycose mutant contained more protein than the parent that fractionated differently. This increased proportion of protein in the CLC glycosylation mutant is likely due to less carbohydrate in the CLC and therefore proportionately more protein.

**Table 2:** Protein concentrations of Triton X-114 and sarkosyl fractions.

***F. tularensis* strain**	**Protein (μg/ml)**
**WbtI**_**G191V**_**_P10**	
TxS-A	352
TxS-D	364
TxI-SS	302
TxI-SI	20
**WbtI**_**G191V**_Δ**1423-22_P10**	
TxS-A	426
TxS-D	392
TxI-SS	775
TxI-SI	330

*TxS, Triton X-114 soluble; TxI, Triton X-114 insoluble; A, aqueous; D, detergent; SS, sarkosyl soluble; SI, sarkosyl insoluble*.

### Impact of growth medium on CLC expression

CLC is normally extracted from bacteria that have been grown for an extended period of time on CDMA. To determine if CLC was enhanced/upregulated under any other growth conditions, WbtI_G191V__P10 was grown in broth and on agar of CDM, BHI, and MH. The CLC and HMS band/smear was highly expressed upon growth of bacteria on CDMA and BHIA, but not MHA (gel electrophoretic data not shown), supporting prior analysis of *F. tularensis* surface antigens following growth on these media (Zarrella et al., 2011; Holland et al., 2017). When *F. tularensis* was grown for 10 days in CDMB or BHIB, CLC was not detected in the broth medium. However, when WbtI_G191V__P10 was grown for 10 days in MHB, material similar to the HMS CLC band/smear appeared to be released into the broth supernatant following centrifugation of the cells (Supplementary Figure 1). When the bacterial cells grown in MHB for 10-days were extracted for CLC using urea, only faint evidence of a band in the region of the HMS band appeared with silver staining. Therefore, the production and/or localization of CLC can be influenced by the growth medium, with both CDM and BHI favoring production and cellular retention of CLC.

### Composition of the CLC

**Figure 4:**
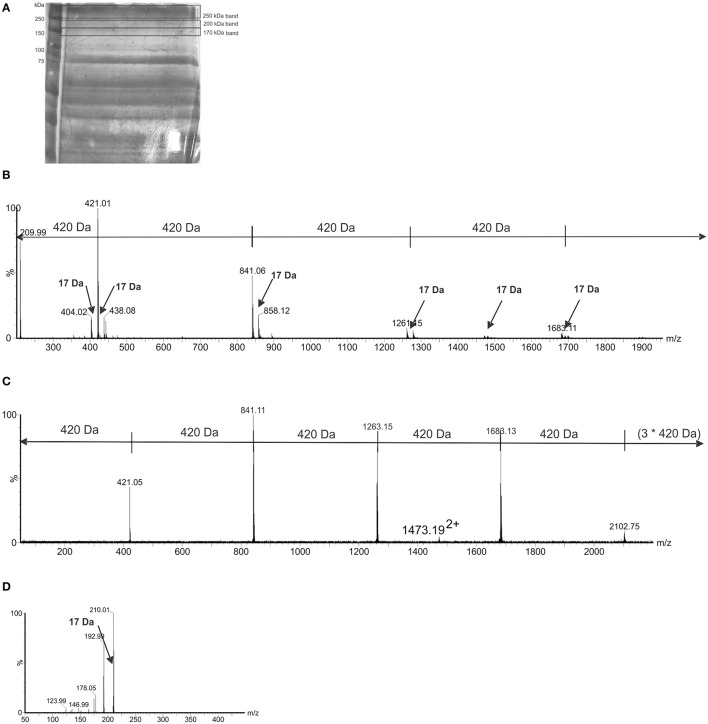
Mass-spectrometry (MS) of high molecular size putative CLC bands excised from LVSΔWbtI_P10 1 M urea extract. **(A)** 8% SDS-PAGE of the CLC extracted from WbtIG191V_P10 CLC with 1 M urea and stained with StainsAll/silver. The extract was concentrated through a 100-kDa filter and bands indicated by black lines were excised and analyzed by MS; **(B)** a LC-MS trace from tryptic digest of the 200-kDa and 170-kDa gel bands showed a 420-Da repeating unit, with the number of 420-Da units/chain lengths being variable. There was evidence of a 420-Da unit in the band/smear >250-kDa, but the longer chains were not observed. In addition to the major ions, observed at 420 Da intervals, minor ions were observed, corresponding to the addition and loss of a 17-Da ammonia molecule; **(C)** a MS/MS of m/z 1682^2+^, showing fragment ions at intervals of 420 Da; D, MS/MS spectrum of 421^+^, an ion putatively corresponding to the submit mass of the observed chains. Fragment ions were observed at m/z 210, 192, 178, 146, and 123. A loss of 17 Da, likely corresponding to ammonia, was observed.

Analysis of the urea-extracted CLC identified a large percentage of acidic and hydrophobic amino acids (Supplementary Table 2), which is indicative of proteins that will self-aggregate and be highly insoluble in aqueous solutions. Mass spectrometry analysis of in-gel digests did not identify any proteins, and manual inspection of the MS/MS spectra showed no evidence of spectra containing characteristic peptide y and b ions. However, the nLC-MS/MS spectra of in-gel tryptic digests of both 200-kDa and 170-kDa bands showed a 420-Da repeating unit (Figures 4A,B). The spectra showed the chains to be variable in length. MS/MS of a doubly charged ion from the 200-kDa MS spectrum also showed fragment ions separated by 420 Da, indicating a chain of seven units of 420 Da. The speculative addition and loss of a 17-Da ammonia residue was also observed in the MS and MS/MS spectra (Figures 4B,C). Target MS/MS of an ion at m/z 421, showed fragment ions at m/z 210, 192, 178, 146, and 123. A loss of 17 Da, likely corresponding to ammonia was observed (Figure 4D). These data suggest that the high molecular weight bands are comprised of chains potentially glycan in nature, with a subunit of 420-Da. There was also evidence of a 420-Da unit in the band larger than 250-kDa, but the longer chains were not observed.

Further analysis of the urea-extracted CLC that was concentrated through a 100-kDa Centriprep filter unit was performed. Following concentration, some CLC material fell out of solution and was recovered by centrifugation at 3000 rpm as the insoluble fraction. The carbohydrate content of the insoluble and soluble fractions of the CLC were similar, yielding just over 10% carbohydrate/mg of CLC (soluble, 112 μg carb/mg CLC; insoluble, 138 μg carb/mg CLC). The predominant sugars identified were glucose, galactose, and mannose, as previously reported (Bandara et al., 2011). However, many samples also contained hydroxylated fatty acids and 3-deox-D-*manno*-2-octulosonic acid (KDO), indicating the presence of outer membrane LPS (data not shown).

**Figure 5:**
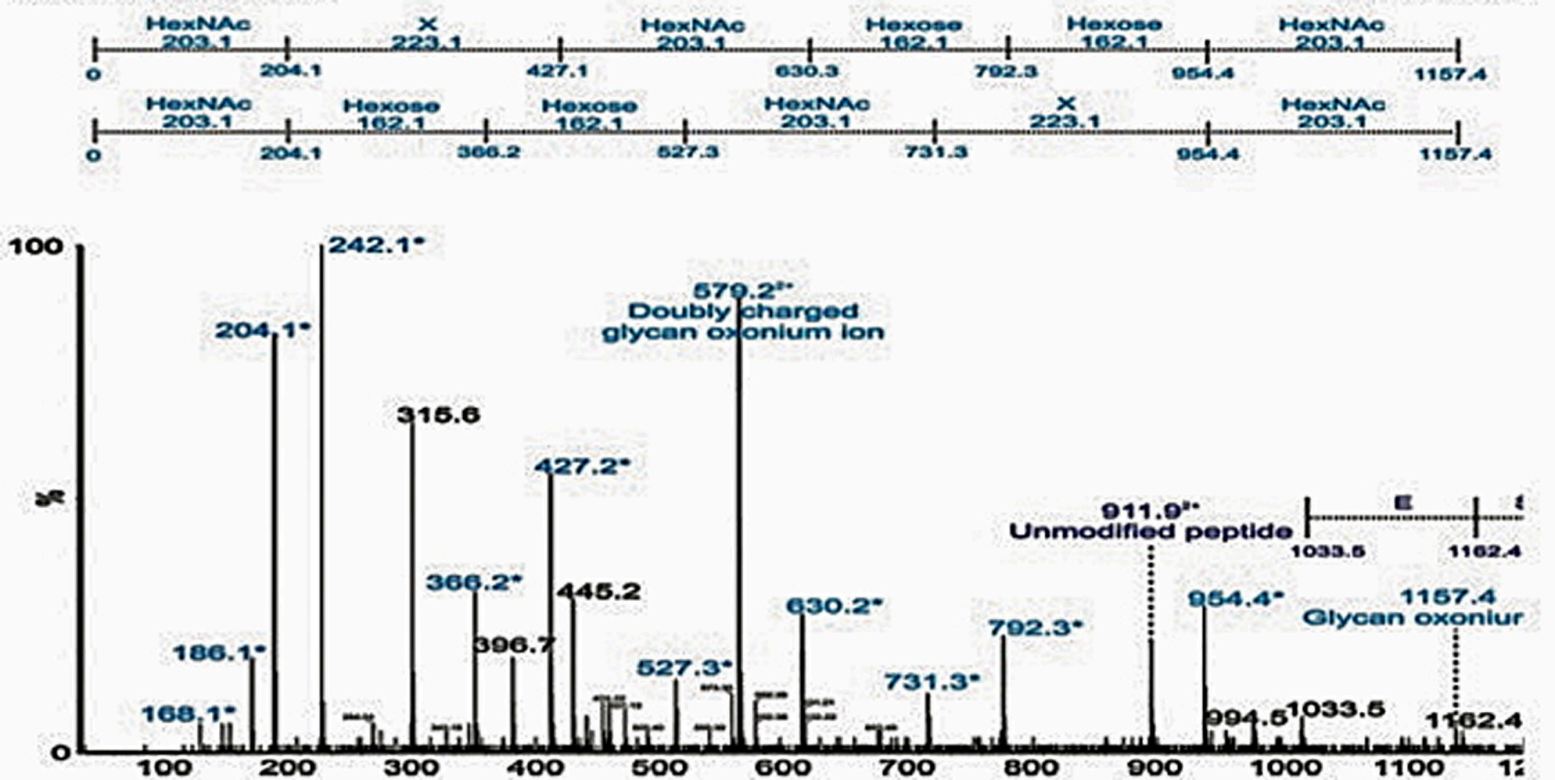
Detection of glycopeptides in the soluble capsule extract. nLC-MS/MS spectrum of a triply protonated glycopeptide at m/z 993.7, enriched from soluble fraction using ion-pairing normal phase liquid chromatography (IP-NPLC). Relative ion abundance is shown on the y axis, and m/z is shown on the x axis. The spectrum is dominated in the low-m/z region by a glycan related ions, indicated in light blue and with an asterisk. In particular, a glycan-related oxonium ion was visible at m/z 1157, corresponding to a previously reported hexasaccharide modification of proteins in *F. tularensis*.

Based on mass spectrometry analysis a total of 68 proteins were identified in the LVS urea-extracted CLC, which were not recovered from the electrophoretic gel. Twelve of these proteins were present in the soluble material (Table 3) and 56 proteins were present in the insoluble material (Table 4). Of interest was that two proteins in the soluble portion of the CLC (chaperone proteins DnaK and GroEL; FTL_1191 and FTL_1714, respectively, were also among the most prominent 20 proteins identified in OMV by Pierson et al. (2011). GroEL was also present in the insoluble portion of the CLC along with 5 other prominent OMV proteins (FTL_1146, FTL_1743, and FTL_1592, FTL_1442, FTL_1772) (Pierson et al., 2011). Overall, of the 12 proteins identified in the CLC soluble fraction, 8 were also present in OMV (FTL_1474, FTL_1907, FTL_0014, and FTL_0227), and of the 56 proteins identified in the CLC insoluble fraction, 38 were also present in OMV (Table 3). Nine proteins identified in the CLC and in OMV/T (noted in Tables 3, 4) have previously been associated with pathogenesis (Pechous et al., 2008). The soluble portion of the CLC, which contains the HMS smear (data not shown), was also digested and analyzed by nLC-MS/MS. No proteins were identified. However, manual inspection of the data showed an MS/MS spectrum dominated by a 1157-Da glycan-related ion. This moiety likely corresponded to the previously reported hexasaccharide modification of proteins in *F. tularensis* (Figure 5) (Thomas et al., 2011). The doubly charged form of this oxonium ion was also observed at m/z 579, from which carbohydrate related fragment ions were observed as neutral losses of monosaccharides in the sequence 203-223-203-162-162-203 or 203-162-162-203-223-203 (total glycan mass is 1156 Da). These masses plausibly represent monosaccharides such as HexNAc (203) and hexose (162). The loss of 223 does not correspond to a known monosaccharide and is indicated as X in Figure 5. A doubly charged ion at m/z 911 likely corresponds to the unmodified form of the peptide, giving a predicted peptide mass of 1,823, corresponding to a total glycopeptide mass of 2,977, less the 1,156 Da hexasaccharide moiety. The intensity of the carbohydrate ions obscured peptide related y and b type fragment ions, making sequencing of the peptide challenging. A short peptide sequence was determined from putative type y ions in the high m/z region of the spectrum, corresponding to the amino acid sequence (Q/K)(I/L)VSE, which was not sufficient to identify the complete peptide.

**Table 3:** Proteins identified in the soluble fraction of CLC from LVS.

**Identifier**	**Proteins previously reported, novel, or among 20 most common (T20):**	**Description**	**Protein score**	**Mass (Da)**	**Peptide_exp**
**Soluble**					
FTL_1191	T20, P,C	Chaperone protein dnaK (heat shock protein family 70 protein)	222	69,140	0.00046
FTL_0009	E, S	Outer membrane protein	122	19,465	8.40E-05
FTL_1474	N	Transcriptional elongation factor	73	17,692	1.50E-07
FTL_0225	S	Protein chain elongation factor EF-Ts	68	30,940	6.70E-07
FTL_1592	N	Acetyl-CoA carboxylase, biotin carboxyl carrier protein subunit	60	16,394	3.40E-06
FTL_1907	N	Cell division protein	52	39,720	2.10E-05
FTL_1461	P, C	Purine nucleoside phosphorylase	50	26,848	2.50E-05
FTL_0113	S	Intracellular growth locus, subunit c	47	22,119	6.60E-05
FTL_0014	N	Single-strand binding protein	43	17,512	0.00014
FTL_1494	P, E, S	hypothetical protein	41	18,147	0.00033
FTL_1714	T20, P, S, C	Chaperone protein, groEL (Yaron S, *E. coli* 2000)	39	57,367	0.00026
FTL_0227	N	Ribosome recycling factor	38	20,540	0.00043

*T20, Top 20 proteins found in F. novicida OMV (Pierson et al., 2011); P, Proteins previously associated with pathogenesis (Pechous et al., 2008); E, Proteins found in exponential phase OMV/T (McCaig et al., 2013); S, Proteins found in early stationary phase OMV/T (McCaig et al., 2013); C, Proteins found in culture filtrate (Konecna et al., 2010); N, Novel to this work*.

**Table 4:** Proteins identified in the insoluble fraction of CLC from LVS.

**Identifier**	**Proteins previously reported, novel, or among 20 most common (T20):**	**Description**	**Protein_score**	**Mass (Da)**	**Peptide_expect**
**Insoluble**					
FTL_1751	E, S	Elongation factor Tu (EF-Tu)	969	43,363	2.00E-05
FTL_1714	T20, P, C	Chaperone protein, groEL	802	57,367	2.90E-06
FTL_1191	P, C	Chaperone protein dnaK (heat shock protein family 70 protein)	394	69,140	1.10E-07
FTL_1907	E, S	Cell division protein	381	39,720	3.10E-08
FTL_0113	N, P	Intracellular growth locus, subunit C	368	22,119	1.30E-05
FTL_0234	E, S	Elongation factor G (EF-G)	250	77,681	2.00E-05
FTL_0112	P, S	Intracellular growth locus, subunit B	226	57,881	2.50E-13
FTL_1795	E, S	ATP synthase beta chain	224	49,834	3.40E-07
FTL_0891	N	Trigger factor (TF) protein (peptidyl-prolyl cis/trans isomerase)	190	49540	1.70E-10
FTL_0094	P, E, S	ClpB protein	178	95,987	1.00E-07
FTL_0225	N	Protein chain elongation factor EF-Ts	177	30,940	1.40E-06
FTL_1146	T20, C	Glyceraldehyde-3-phosphate dehydrogenase	161	35,391	0.0003
FTL_0267	P, S	Chaperone Hsp90, heat shock protein HtpG	151	72,326	1.00E-07
FTL_1791	N, C	Superoxide dismutase	148	21,926	9.30E-07
FTL_1912	E, S	30S ribosomal protein S1	145	61,631	0.00017
FTL_1461	N, C	Purine nucleoside phosphorylase	132	26,848	0.0007
FTL_0436	N	Isoleucyl-tRNA synthetase	127	106,904	6.00E-13
FTL_0572	E, S	Hypothetical protein	113	51,945	1.10E-11
FTL_0260	S	30S ribosomal protein S4	107	23,222	8.10E-11
FTL_0597	N	NAD dependent epimerase	105	36,427	1.80E-07
FTL_0895	N, C	Histone-like protein HU form B	105	9,468	1.20E-10
FTL_1190	N	Chaperone protein grpE (heat shock protein family 70 cofactor)	102	22,022	2.50E-06
FTL_0166	S	Universal stress protein	102	30,202	2.10E-06
FTL_1746	S	50S ribosomal protein L10	101	18,720	1.90E-10
FTL_0949	N	Ribose-phosphate pyrophosphokinase	97	34,887	2.70E-06
FTL_0987	S	Lactate dehydrogenase	94	34,055	5.90E-07
FTL_0588	N	Isocitrate dehydrogenase	93	82,332	7.70E-05
FTL_0834	S	Rhodanese-like family protein	88	27,847	4.90E-09
FTL_1553	P, S	Succinyl-CoA synthetase beta chain	87	41,516	1.30E-06
FTL_1527	S	Enolase (2-phosphoglycerate dehydratase)	85	49,480	4.30E-09
FTL_1701	P	GlpX protein	83	34,820	1.90E-08
FTL_0923	S	Glutaredoxin 2	83	25,135	1.30E-08
FTL_1224	N	Thioredoxin 1	78	12,194	6.60E-08
FTL_1743	T20,P, S,E	DNA-directed RNA polymerase, β subunit	77	157,289	6.20E-08
FTL_1591	S	Acetyl-CoA carboxylase, biotin carboxylase subunit	76	50,019	7.50E-08
FTL_0097	N	Hypothetical protein	76	13,785	6.10E-05
FTL_1780	N, C	Triosephosphate isomerase	76	27,638	5.80E-08
FTL_0232	S	30S ribosomal protein S12	69	13,806	9.70E-07
FTL_1350	N	Glycyl-tRNA synthetase beta subunit	66	77,973	4.80E-07
FTL_1140	N	Malonyl coA-acyl carrier protein transacylase	62	33,480	2.30E-06
FTL_0233	S	30S ribosomal protein S7	61	17,796	2.60E-06
FTL_1138	N	acyl carrier protein	61	10,653	2.30E-06
FTL_1240	S	Phospho-2-dehydro-3-deoxyheptonate aldolase	60	40,853	1.80E-06
FTL_1747	S	50S ribosomal protein L1	57	24,626	4.70E-06
FTL_1275	N	Dethiobiotin synthetase	55	24,487	9.70E-06
FTL_1592	T20	Acetyl-CoA carboxylase, biotin carboxyl carrier protein subunit	53	16,394	1.60E-05
FTL_1334	N	L-serine dehydratase 1	52	49,977	2.70E-05
FTL_0387	N	Aspartate transaminase	52	44,355	2.60E-05
FTL_1442	T20, S	Enoyl-[acyl-carrier-protein] reductase (NADH)	49	27,757	4.10E-05
FTL_0916	E, S	Ketol-acid reductoisomerase	46	37,855	7.30E-05
FTL_1797	N	ATP synthase alpha chain	46	55,502	5.20E-05
FTL_0311	S	Dihydrolipoamide dehydrogenase	44	50,495	9.90E-05
FTL_1772	T20, S	Aconitate hydratase	44	102,639	0.00028
FTL_1784	E	2-oxoglutarate dehydrogenase E1 component	39	105,613	0.00029
FTL_0739	N	Glucose inhibited division protein A	36	69,721	0.00053
FTL_1022	N	Coproporphyinogen III oxidase	36	35,854	0.00053

*T20, Top 20 proteins found in F. novicida OMV (Pierson et al., 2011); P, Proteins previously associated with pathogenesis (Pechous et al., 2008); E, Proteins found in exponential phase OMV/T (McCaig et al., 2013); S, Proteins found in early stationary phase OMV/T (McCaig et al., 2013); C, Proteins found in culture filtrate (Cervenka et al., 2010). N = Novel to this work*.

### Comparison of OMV/T with CLC

**Figure 6:**
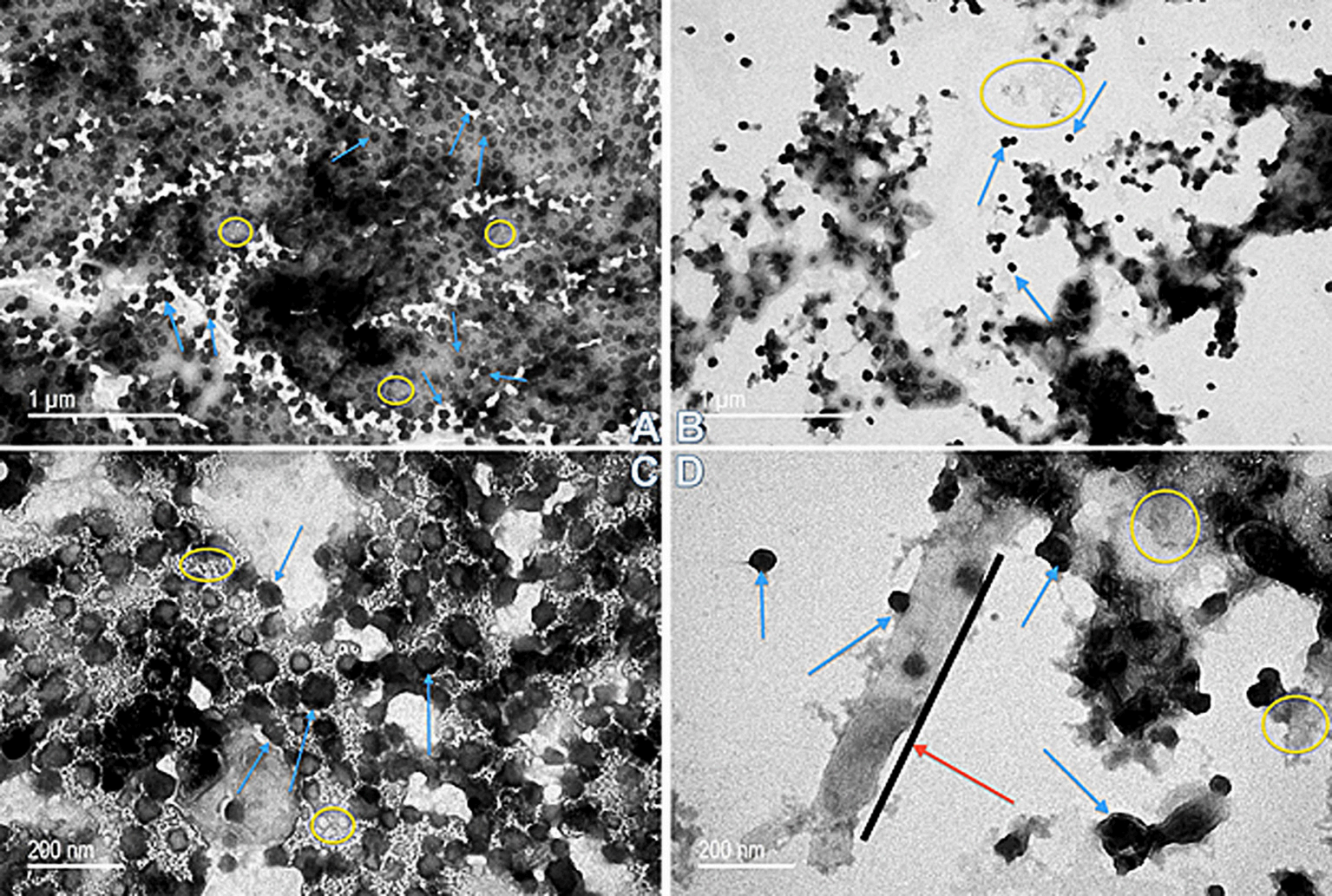
Transmission electron microscopy of OMV/T purified from *F. tularensis* strains grown in broth**. (A)** OMV/T from WbtI_G191V__P10 (50 k magnification); **(B)** WbtI_G191V_Δ1423-22_P10 (50 k magnification); **(C)** WbtI_G191V__P10 (120 k magnification); **(D)** WbtI_G191V_Δ1423-22_P10 (120 k magnification). Spherical OMV were found in both the O-antigen mutant (WbtI_G191V__P10) and, to a lesser extent, the CLC glycose-deficient double mutant (WbtI_G191V_Δ1423-22_P10). At the higher magnification formation of OMT were visible by WbtI_G191V_Δ1423-22_P10 in addition to OMV, which was not commonly seen in the O-antigen mutant. Red arrows/black lines, OMT; blue arrows, OMV; yellow circles, non-specified material between OMV/T.

**Figure 7:**
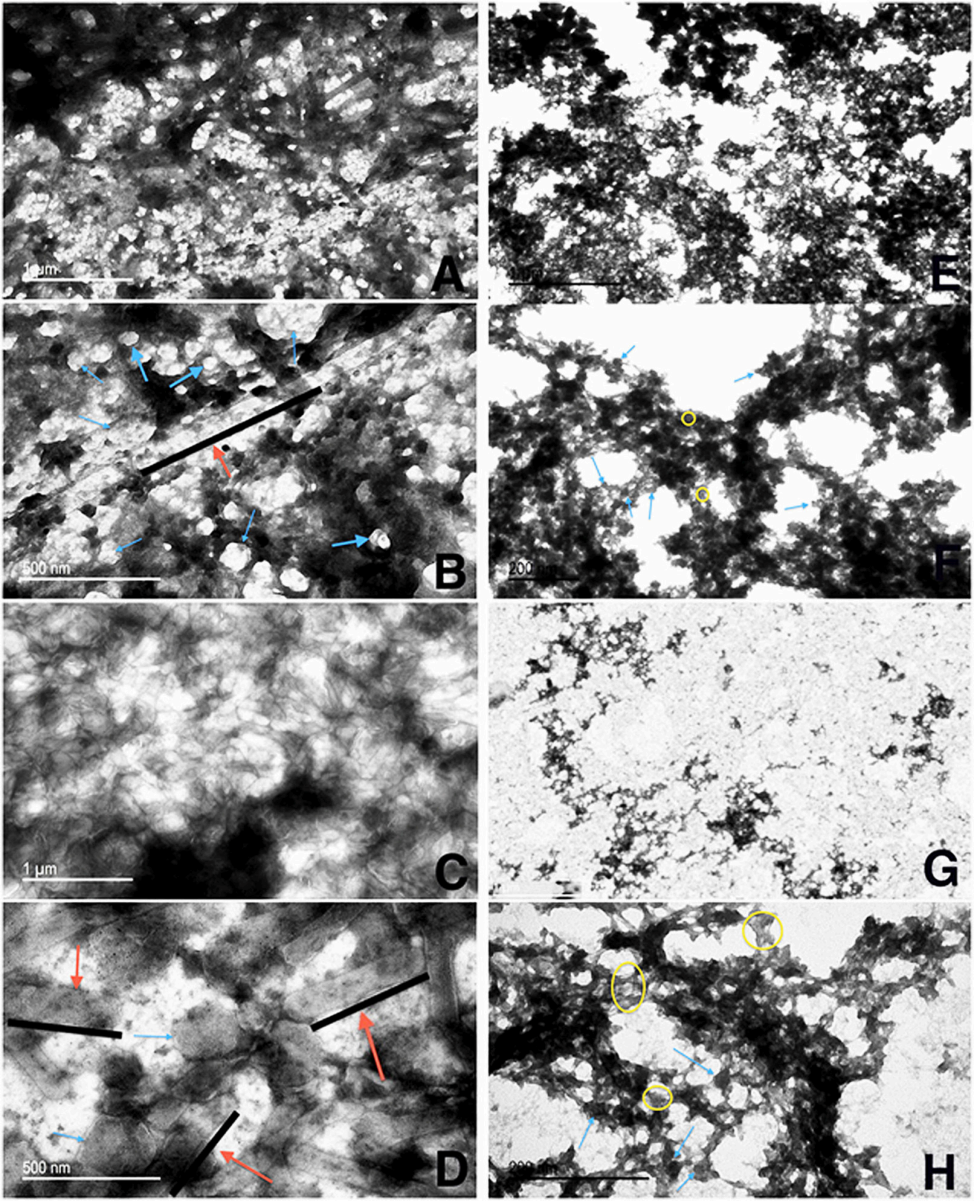
Transmission electron microscopy of OMV/T_A_ purified from *F. tularensis* strains grown on agar. OMV/T_A_ were isolated from LVS_P10, LVSΔ1423-22_P10, WbtI_G191V__P10, or WbtI_G191V_Δ1423-22_P10 grown on CDM agar following a modified protocol (Gamazo et al., 1989; Avila-Calderón et al., 2012). **(A,B)** LVS_P10 (50 k magnification and 120 k magnification, respectively); **(C,D)** LVSΔ1423-22_P10 (50 k magnification and 120 k magnification, respectively); **(E,F)** WbtI_G191V__P10 (50 k magnification and 120 k magnification); **(G,H)** WbtI_G191V_Δ1423-22_P10 (50 k magnification and 120 k magnification, respectively). Red arrows, OMT_A_ (along black lines); blue arrows, OMV_A_; yellow circles, non-specified material between the OMV/T_A_.

Because the CLC appeared to share many proteins with those previously identified in OMV, further comparison of OMV/T to CLC was carried out. There were distinct differences in the shape and type of OMV/T observed when OMV/T were extracted from cells grown in broth or on agar. When OMV/T were extracted from either mutants WbtI_G191V__P10 or WbtI_G191V_Δ1423-22_P10 grown in broth medium, the vesicles were predominately circular in appearance (Figures 6A–D, respectively), including LVS_P10 and LVSΔ1423-22_P10 (not shown), although some tubes were present, particularly from WbtI_G191V_Δ1423-22_P10. When the OMV/T_A_ were extracted from bacteria grown on solid agar, the particles from LVS_P10 had an approximately even distribution of circular vesicles and tubes (Figures 7A,B), while the OMV/T_A_ from LVSΔ1423-22_P10 (CLC glycosylation-deficient mutant) were predominantly tubular in shape (Figures 7C,D). When both O-antigen deficient mutants WbtI_G191V__P10 (Figures 7E,F) and its CLC glycose-deficient double mutant WbtI_G191V_Δ1423-22_P10 (Figures 7G,H) were grown on solid agar, only circular OMV_A_, but not tubes, were seen. Therefore, when the bacteria were grown in broth the vesicles were predominately circular, regardless of whether the CLC was glycosylated or O-antigen was present. However, growth of the bacteria on agar promoted OMT_A_ formation by LVS_P10 (Figures 7A,B), but the CLC glycosylation mutant of the parent formed even more tube-shaped vesicles (Figures 7C,D). Mutants grown on agar that lacked O-antigen produced predominately circular vesicles (Figures 7E,F), but if the mutant lacked both O-antigen and CLC glycosylation there was overall less OMV/T_A_ produced (Figures 7G,H). Therefore, the presence of O-antigen and/or CLC glycosylation also influenced the shape and amount of OMV/T_A_ produced.

There was a significant size difference (*p* < 0.0055) of both the OMV_A_ and the OMT_A_ from LVS_P10 and LVSΔ1423-22_P10 compared to WbtI_G191V__P10 and WbtI_G191V_Δ1423-22_P10 following growth of all strains on CDMA (Figure 7). The average size of the OMV_A_ extracted from LVS_P10 (Figures 7A,B) ranged from 20 to 200 nm in diameter, while the average OMV_A_ extracted from WbtI_G191V_ was 10–40 nm in diameter (Figures 7E,F). The average size of the OMT_A_ extracted from plate-grown LVS_P10 was much larger than the vesicular form, with an average length of 500 nm, which was equivalent to the length of OMT occasionally seen in the WbtI_G191V_ strain. Therefore, the presence of O-antigen and growth on agar also promoted formation of larger OMV/T_A_.

Interestingly, protein concentrations of purified OMV/T increased in the CLC-deficient mutants in both strains when the bacteria were grown in broth, but when the bacteria were grown on agar (as done for CLC-extraction), the protein content decreased in the WbtI_G191V_Δ1423-22_P10 mutant compared to WbtI_G191V_ (Table 2).

**Figure 8:**
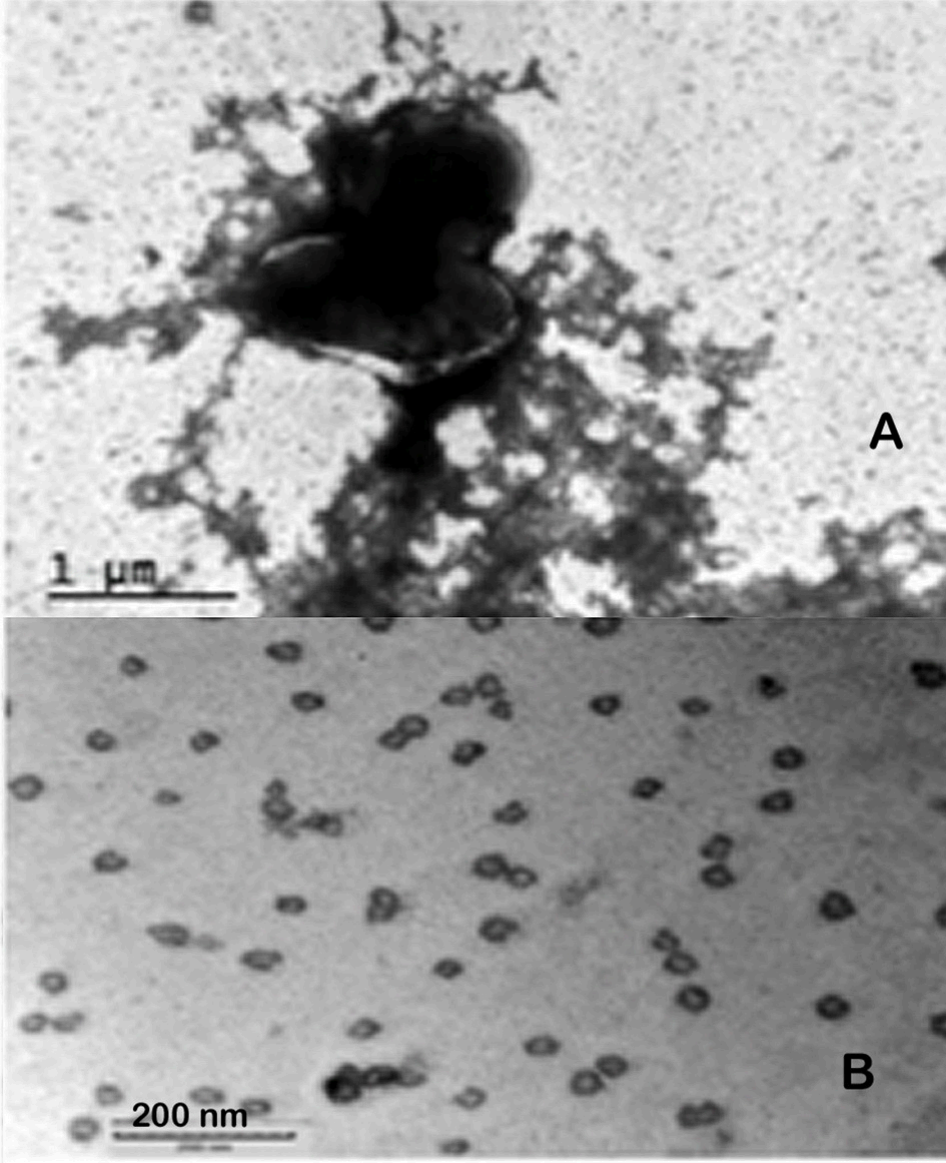
TEM of *F. tularensis* WbtI_G191V_ enhanced for CLC expression, and CLC extracted with urea. **(A)** Whole bacteria surrounded by CLC; **(B)** urea-extracted CLC. Whole cells were fixed with glutaraldehyde before staining and all samples were negatively stained with 0.5% uranyl acetate.

When WbtI_G191V__P10 was examined by negative stain TEM the typical electron dense material was present surrounding the bacterial cell (Figure 8A), which appeared similar to low power TEM of OMV_A_ (Figure 7). However, following higher magnification of the urea-extracted CLC, individual circular vesicles were observed (Figure 8B) that were similar in ultrastructural appearance to OMV.

### Protective efficacy of CLC

**Figure 9:**
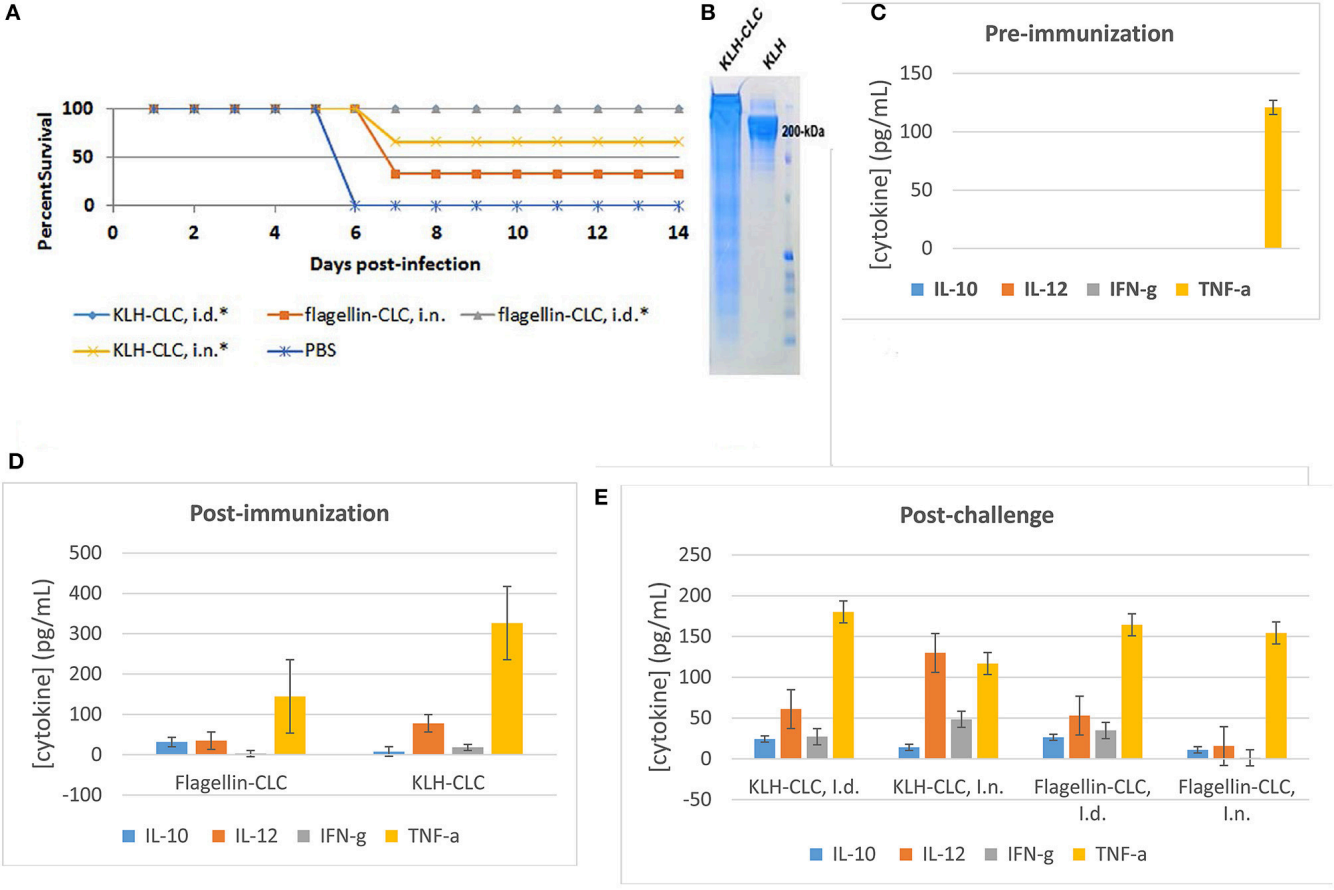
Protective efficacy of CLC-conjugate in BALB/c mice challenged intradermally and intranasally, and cytokine response pre- and post-challenge. **(A)** Survival of BALB/c mice challenged ID or IN with 5 × LD_50_ of LVS after ID immunization with 50 μg of KLH- or flagellin-conjugated CLC. Mice immunized with either conjugate were significantly protected against ID challenge, and mice immunized with KLH-CLC were protected against IN challenge (^*^p < 0.05). **(B)** SDS-PAGE of conjugated KLH-CLC and unconjugated KLH, showing the increase in size of the conjugate. **(C)** Pre-immunization serum cytokine response of all mice; **(D)** cytokine response from sera of mice collected 2 weeks after the final immunization with KLH-CLC or flagellin-CLC; **(E)** cytokine response from sera collected 3 days post-challenge.

The CLC was conjugated to either KLH or flagellin because the material was initially believed to be predominately polysaccharide. Conjugation of CLC to either protein was confirmed by SDS-PAGE, in which the conjugated CLC appeared as a band/smear above 200-kDa (Figure 9B). The CLC-conjugate provided some protection against lethal LVS challenge of BALB/c mice. However, protection of mice challenged by the IN route was less effective than by the ID route. Mice immunized with the flagellin-CLC conjugate, followed by IN challenge, were the least protected (33% of mice survived) (Figure 9A). Protection was improved in mice immunized with CLC conjugated to KLH (66%) after IN challenge (one-sided *p* = 0.03). Only mice challenged by the ID route were completely protected (100%) against mortality when immunized with either KLH—or flagellin-conjugates (one-sided *p* = 0.001). Results of the mice immunized with KLH-CLC are superimposed with results of mice immunized with flagellin-CLC (Figure 9A). However, the LVS challenge dose was severe at 5 × LD_50_, which was ~5000 CFU for IN and ~2x10^7^ CFU for ID challenge.

Sera were collected from immunized BALB/c mice prior to immunization, post-immunization, and post-challenge for cytokine analysis. There was no significant difference in the response to cytokines IL-2, IL-4, IL-5, GM-CSF, or TNF-α by BALB/c mice immunized by either route with CLC-protein conjugates after challenge with LVS compared to mice immunized with PBS or pre-immunization (data not shown except for TNF-α). However, IL-12 levels were significantly increased in both KLH-CLC and flagellin-CLC immunized mice, compared to mice immunized with PBS and prior to immunization (Figures 9C,D) (*p* < 0.001). Mice that made a significantly greater response to IL-10, IL-12, or IFN-γ after challenge (*p* < 0.001) were better protected than control mice in which that response did not occur. Of interest, the IL-10, IL-12, and IFN-γ response of mice immunized with flagellin-CLC was not significantly increased following IN challenge, and these mice were not significantly protected (Figures 9A,E).

Mice immunized with non-conjugated CLC in Freund's or MPLA adjuvants (as described in Methods) and challenged IN with 100 or 150 CFU of SCHU S4 were not significantly protected (Table 5). There was a time delay to morbidity and mortality by 4-6 days in a few mice immunized 3 months apart compared to control mice, but no significant difference in survival.

**Table 5:** Protective efficacy of *F. tularensis* type A CLC against type A SCHU S4 challenge of mice.

**Number of mice**	**Immunogen**	**Challenge dose**	**Time to euthanasia post-challenge (PC)**
4	Water^a^	100 CFU	4/4 at 4 days PC
8	Crude CLC in FA^a^^,^^b^	100 CFU	4/4 at 4 days PC
8	HMS CLC in FA^a^^,^^c^	100 CFU	4/4 at 4 days PC
5	HMS CLC in MPLA^a^^,^^d^	100 CFU	4/4 at 4 days PC
5	HMS CLC in FA^e^^,^^b^	150 CFU	3/5 at 1 day PC; 2/5 at 5 days PC
5	HMS CLC in MPLA^e^^,^^d^	150 CFU	4/5 at 4 days PC; 1/5 at 7 days PC

a *Mice were immunized subcutaneously with 50 μg of CLC twice 6 weeks apart*.

b *FA, Freund's Adjuvant, first immunization was with Complete Adjuvant and the second immunization was with Incomplete Adjuvant. Crude CLC was derived from WbtI_G191V__P10 cells that had been extracted with 1 M urea and the bacterial cells removed by centrifugation*.

c *HMS CLC, semi- purified CLC including the high molecular size surface antigen was concentrated*.

d *MPLA, Monophosphoryl lipid A adjuvant*.

e *Mice were immunized twice 3 months apart*.

### The Type A capsule-like complex

**Figure 10:**
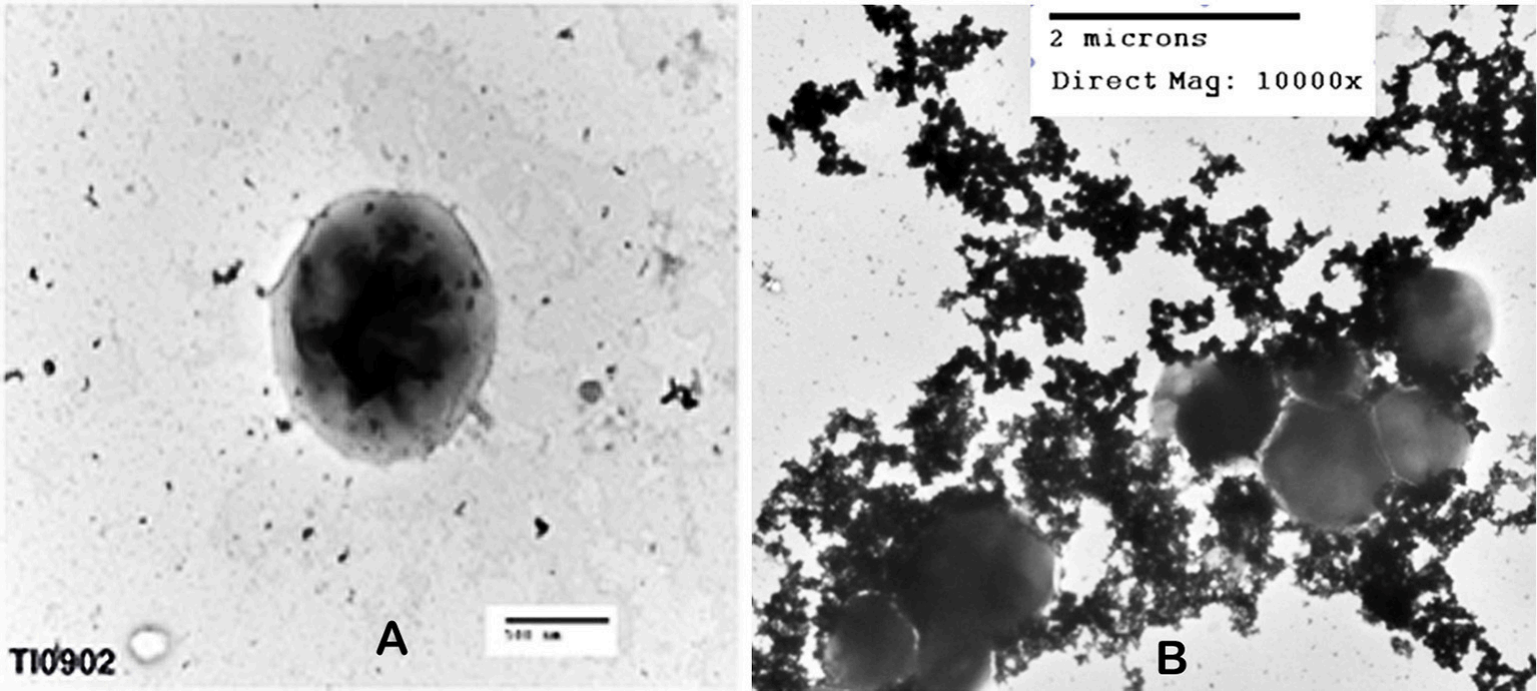
TEM of *F. tularensis* type A clinical isolate strain TI0902. The bacteria were fixed in glutaraldehyde buffer overnight and stained with 0.5% uranyl acetate. **(A)** Strain TI0902 following growth in BHI broth with rapid shaking. **(B)** Strain TI0902 subcultured daily for10 days in CDM broth followed by growth at 32°C for several days on CDM agar, and demonstrating the CLC. Similar CLC material was observed surrounding CDM subcultured type A strain SCHU S4 (not shown). A representative image of the CLC-enhanced O-antigen mutant of strain LVS is shown in Figure 8.

**Figure 11:**
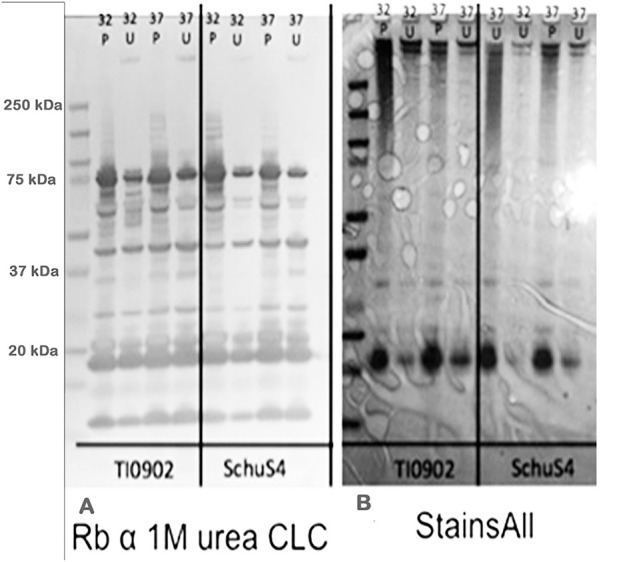
Western blot and electrophoretic profiles of CLC extracted from type A strains TI0902 and SCHU S4 by phenol or urea at 32 or 37°C. **(A)** Western blot of rabbit serum to urea-extracted LVS CLC with CLC extracted from type A strains TI0902 and SCHU S4. **(B)** StainsAll stain of CLC extracts from strains TI0902 or SCHU S4. P, CLC extracted with phenol; U, CLC extracted with urea. The type A strains were subcultured daily in CDMB for 10 consecutive days and then grown on CDMA at 32°C for an additional 5 days.

Colonies of *F. tularensis* type A strains SCHU S4 and clinical isolate TI0902 were more mucoid and iridescent when subcultured in CDMB and grown at 32 or 37°C on CDMA than when these strains were not subcultured. These results were similar to those obtained when LVS (Bandara et al., 2011) and *F. novicida* (Freudenberger Catanzaro et al., 2017) were similarly subcultured in CDMB and CDMA to enhance CLC expression. However, initial efforts to visualize the CLC on the surface of type A strains using negative stain TEM techniques were less successful than with LVS or *F. novicida*. We concluded this was due to the extended period of time the bacteria needed to remain in fixative and at 4°C to verify that no viable cells remained in the culture and were safe to remove from the BSL-3 laboratory. With slight modifications we were able to visualize the darker staining electron dense material surrounding both SCHU S4_P10 (not shown) and TI0902_P10 cells (Figure 10B) that was greatly diminished in the parent that was not subcultured in CDMB and CDMA (Figure 10A). The CLC from the type A strains appeared very similar to that observed around LVS (Figure 8A). Following electrophoresis and StainsAll/silver stain of the CLC a HMS smear, in addition to some lower molecular size bands, were prominent in phenol-extracted CLC from strains SCHU S4 and TI0902 subcultured in CDMB and CDMA (Figure 11B). The urea-extracted type A CLC presented a less diffuse HMS band/smear by SDS-PAGE, similar to LVS CLC, and also included the additional lower molecular size bands. In addition, the type A CLC antigens were immunologically similar to the LVS CLC (phenol and urea extractions; Figure 1A) and reacted with hyper-immune rabbit serum to LVS CLC (Figure 11A). Qualitatively, the electrophoretic profile of the CLC extracts were similar whether the cells were grown on CDMA at 32 or 37°C. However, quantitatively there appeared to be more proteins, particularly the larger molecular size proteins, in the CLC extracted from cells grown at 32°C. Carbohydrate analysis of purified CLC from *F. tularensis* SCHU S4 yielded similar results to the LVS CLC: there was about 5–10% carbohydrate, which consisted of glucose, galactose, and mannose (data not shown).

Mutagenesis of FTT_0798-99 resulted in a significant (*p* < 0.05) loss of carbohydrate on SCHU S4Δ0798-99 (16.39 mg carbohydrate/g bacterial weight compared to 28.57 mg carbohydrate/g bacterial weight for SCHU S4). However, all mice inoculated IN with 100 CFU of SCHU S4 or SCHU S4Δ0798-99 died or needed to be euthanized in <6 days (data not shown). Furthermore, mice challenged with the mutant did not live longer than mice challenged with the parent. Therefore, inactivation of the CLC glycosylation locus had no significant effect on the virulence of SCHU S4, as mutagenesis of the corresponding genes FTL_1423 and FTL_1422 did in LVS (Bandara et al., 2011).

## Discussion

The CLC of *F. tularensis* remains a novel, elusive antigen, shown to be necessary for virulence in LVS and *F. novicida* (Bandara et al., 2011; Freudenberger Catanzaro et al., 2017). However, isolation and analysis of the CLC has been challenging due to self-aggregation and insolubility of the CLC during purification. By changing the chaotropic extraction buffer to 1 M urea a marked increase in solubility and ease of manipulation was obtained, but confirmation that extraction of the CLC with 1 M urea resulted in the same antigen extracted with 0.5% phenol (Bandara et al., 2011) was necessary. SDS-PAGE electrophoretic profiles of the CLC extracted by phenol or urea and stained with StainsAll/Silver showed that these antigens were similar, though the banding pattern of the HMS was more diffuse following phenol compared to urea extraction. The more diffuse banding was likely the result of protein denaturation by the phenol. Western blot electrophoretic profiles using immune sera generated to WbtI_G191V__P10 CLC extracted with either phenol or urea and types A and B CLC antigens extracted by either reagent were very similar. However, the larger molecular size bands apparently did not transfer efficiently out of the gel. Nonetheless, the CLC antigens from types A and type B strains were similar, regardless of the extraction method.

The individual components of the HMS material have been difficult to resolve. If the HMS CLC is an aggregate of glycoproteins or a mixture of (glyco)proteins and polysaccharide, individual components should be resolved using a size exclusion system based on electrophoretic mobility. The GelFree 8100 cartridge is capable of fractionating components up to 500-kDa. However, the largest CLC band visualized by silver stain was just under 150-kDa after fractionation, and no bands or diffuse HMS material was observed with StainsAll/Silver. Thus, the HMS CLC observed was likely the result of aggregates of CLC (glyco)-proteins, which were separated into individual components during GelFree fractionation. The 45-kDa band that appeared in the highest molecular size GelFree fractions (>90-kDa) was also the only component reactive after hyper-immune serum to CLC after adsorption with CLC-deficient mutant cells. Therefore, the 45-kDa protein could be involved in the aggregation of the CLC. Furthermore, the 45-kDa protein has been recognized as an immuno-reactive glycoprotein in mouse sera following immunization with OMP's (Huntley et al., 2008). Although identification of this band was unsuccessful following concentration of the fractions and mass spectrometry (likely due to glycosylation) this protein was similar in size to outer membrane protein FopA, which is also glycosylated, highly immunogenic, and potentially protective (Hickey et al., 2011).

To further increase the solubility and characterize the CLC, extraction with Triton X-114 was tested, which was previously used to purify the *F. tularensis* O-antigen capsular polysaccharide (Apicella et al., 2010). However, Triton-X114-insoluble material could be further extracted into soluble components with sarkosyl. The total CLC protein solubilized with Triton X-114 fractionated relatively equally between the TxS-A phase and TxS-D phase. However, the HMS material from the O-antigen mutant partitioned primarily into the TxS-A phase, as determined by SDS-PAGE. These results suggested that the CLC consisted of both hydrophilic and hydrophobic components, such as glycoproteins (that may partition into the hydrophilic TxS-A phase), and integral membrane proteins, OMP, LPS, and lipoproteins (that may partition into the TxS-D phase), as is well-documented (Bordier, 1981; Tandon et al., 1989). Partitioning of the HMS into TxS-A is also consistent with a larger amount of hydrophilic carbohydrate in this material. Most of the CLC extracted from glycose-deficient mutant WbtI_G191V_Δ1423-22_P10 was not solubilized in Triton X-114. However, the TxI-SS fraction of the double mutant did demonstrate a diffuse, darker staining HMS material by SDS-PAGE and silver staining, indicating the mutant CLC contained different proteins or glycose-deficient proteins than the parent CLC. We have previously reported that WbtI_G191V_Δ1423-22_P10 (Bandara et al., 2011), and an *F. novicida* mutant lacking genes from the same glycosylation locus (Freudenberger Catanzaro et al., 2017), are deficient in, but continue to produce, a lesser amount of carbohydrate-deficient CLC. The difference in protein content between the O-antigen deficient parent and the glycose-deficient CLC mutant was evident in the ratio of TxI-SS:TxI-SI fractions. The CLC from WbtI_G191V__P10 had a protein ratio of 15:1 (TxI-SS:TxI-SI) vs. 2:1 for CLC from the double, glycose-deficient mutant. In support of our previous report (Freudenberger Catanzaro et al., 2017), mutagenesis of the glycosylation locus alone does not appear to affect protein content, but does affect association with the cell surface, which does affect recovery of the CLC.

We have previously reported that the CLC could be extracted from *F. tularensis* cells subcultured in CDMB and then grown for several days on CDMA at 32°C, but could not be recovered from the broth medium of shaken cultures (Bandara et al., 2011). We now confirm those results, and that the CLC can also be recovered from *F. tularensis* cells grown on BHIA, and at 37°C, but not from MHA. However, *F. tularensis* WbtI_G191V__P10 shed some HMS material, similar to what was recovered by CLC extraction, when the bacteria were grown in MHB for 10 days. One possibility is that the HMS material shed during growth in MHB is part of the CLC, but remains attached to the cell when grown on CDMA. The MH broth-shed HMS material was also similar to the HMS carbohydrate previously described (Zarrella et al., 2011). This HMS carbohydrate is “missing” during bacterial growth in MHB, but is present when the bacteria are grown in BHI, which is proposed to mimic the bacterial environment during host infection. Nonetheless, the growth media CDM and BHI influenced production and retention of the CLC on the cell surface.

A large number of *Francisella* surface proteins are known to be glycosylated, including pili, DsbA, FTH_0069, FopA, Tul4, LemA, and others (Balonova et al., 2010). Many of the CLC proteins were also glycosylated, as evidenced by staining of electrophoretically separated glycoproteins with StainsAll and by chemical analysis. Further evidence of glycosylation was supported by the presence of a 420-Da repeating unit of the HMS material, which is most likely a repeating glycan unit. An amino acid analysis of the CLC indicated the proteins consisted predominately of acidic or hydrophobic amino acids, which would promote self-aggregation and aqueous insolubility of the CLC. Many of the 56 proteins in the CLC extracted from WbtI_G191V__P10 were surface proteins and proteins involved in virulence (including some FPI proteins). Sixty-percent of these proteins have also been identified in the OMV of *F. novicida* (McCaig et al., 2013). Proteins identified previously in *Francisella* OMV and OMV/T have numbered in the hundreds (Pierson et al., 2011; McCaig et al., 2013). However, the CLC extract analyzed in this study consisted of substantially fewer proteins, which may be due to the extraction methods used during the purification process. In addition to similarities in protein composition, GC-MS analysis confirmed that while the predominant sugars in the CLC were glucose, galactose, and mannose, LPS components (KDO and lipid A fatty acids) were also in many of the samples analyzed, supporting the presence of outer membrane components. There was also TEM ultrastructural similarity between CLC particles isolated with urea and OMV from *F. novicida* and *F. philomiragia* (Pierson et al., 2011).

The shape and size of OMV/T isolated from *F. tularensis* was also dependent on whether the bacteria were grown in broth or on agar (larger tubes), and whether the LPS O-antigen was present and the CLC glycosylated. O-antigen mutants generally made smaller vesicles, and the CLC glycose-deficient mutants were predominately tubular in shape. However, from bacteria with both O-antigen and CLC carbohydrate absent, much fewer OMV/T were observed. Therefore, glycosylation, which may promote a more hydrophilic surface, may promote more of the circular vesicle shape and more OMV in general.

An effective vaccine for *F. tularensis* is proposed to require both cell-mediated and humoral immunity (Elkins et al., 2007; Eyles et al., 2008; Kirimanjeswara et al., 2008; Reed et al., 2014). The *F. tularensis* OMP and OMV/T have been shown to induce a pro-inflammatory response by the host (Huntley et al., 2008; McCaig et al., 2013), which also occurs following immunization with the CLC. Immunization of mice with the CLC produced an increase in pro-inflammatory cytokines, particularly IL-12 and IFN-γ. In the BALB/c mouse model, CLC conjugated to an immunogenic protein was protective against lethal LVS challenge. One hundred percent survival was obtained using either KLH or flagellin as the conjugative protein when mice were challenged ID with LVS. Significant protection against *F. novicida* IN challenge has also been established using OMV, but the protection was incomplete and did not lead to 100% survival of OMV-vaccinated mice (Pierson et al., 2011). However, a vaccine for tularemia needs to protect against the aerosol route of infection by the more virulent type A strains. BALB/c mice immunized by the ID route with type A CLC and Freund's Complete adjuvant were not protected against IN challenge with a high lethal dose of *F. tularensis* SCHU S4. The challenge does of SCHU S4 given to the mice (500 CFU) in the present study was ~10 times higher than the challenge dose given by Huntley *et. al*. who reported a 50% survival rate following immunization of mice with a mixture of OMPs (Huntley et al., 2008). Differences in the preparation of OMPs compared to CLC and differences in challenge dose could account for the differences in protection between studies. Immunization of mice with CLC derived from type A strain SCHU S4 did not protect mice against high dose IN challenge with SCHU S4. Therefore, CLC alone would not be adequate for a type A tularemia vaccine, but could have potential as a component of a subunit vaccine, or may protect against a lower challenge dose of virulent type A *F. tularensis*.

Deletion of the LVS glycosylation genes FTL_1423 and FTL_1422 significantly decreased the CLC material (*p* = 0.01) and attenuated the strain in mice (Bandara et al., 2011). Thomas et al. reported that deletion of the FTL_1423 homolog FTT_0798 resulted in the loss of an O-linked 1156-Da glycan moiety on type A *F. tularensis* DsbA, but virulence was not affected (Thomas et al., 2011). Our work supported this observation in that deletion of FTT_0798 and FTT_0799 also did not adequately attenuate type A strain SCHU S4, at least not against high dose respiratory challenge, although there was a similar loss of cell carbohydrate content.

## Conclusion

We present evidence that the CLC appears similar to OMV/T in appearance and composition, and therefore over-expression of OMV/T in *F. tularensis* type A and B strains may contribute to the presence of CLC. This evidence is based on: (i) the CLC appears similar to OMV/T by TEM, and both CLC and OMV/T are differentially expressed depending on O-antigen and CLC (glycosyl transferase) deficiencies, and (ii) ~60% of the proteins identified in the CLC matched previously identified OMV/T proteins in *F. novicida*, and (iii) the CLC contains some components that are consistent with outer membrane content. Alternatively, it is also possible that CLC preparations are contaminated with OMV/T, or that OMV/T preparations also contain CLC (e.g., the non-specific material highlighted by yellow circles in Figures 6, 7). Contaminating proteins or other extracellular material have been noted to be present in OMV preparations from Gram-negative bacteria derived by ultracentrifugation (Bauman and Kuehn, 2006; Chutkan et al., 2013). Whether OMV/T are integral components of the *F. tularensis* CLC, or the CLC is a contaminant of OMV/T preparations warrants further investigation. A glycose-deficient CLC type A mutant was not attenuated in mice and the CLC was not protective against high-dose type A IN challenge of mice. However, differential expression of CLC, OMV, and OMT appears to be connected, and more insight into their interrelationship could help elucidate novel virulence mechanisms of *F. tularensis*.

## Ethics statement

This study was carried out in accordance with the recommendations of Virginia Tech's Animal Welfare Assurance policy, as described in A-3208-01 (expr. 7-31-2021) and USDA-APHIS-AC Registration Certificate # 52-R-0012 (expr. 10-01-2018) by the Virginia Tech Institutional Animal Care and Use Committee. The protocol was approved by the Virginia Tech Institutional Animal Care and Use Committee.

## Author contributions

ST and KF performed the amino acid sequencing and glycoprotein identification. Mutants used were constructed by AB and NM. All other procedures were performed by AC unless otherwise noted in text. Experimental design was conceived by AC and TI. AC and TI wrote and edited the majority of the manuscript. AB, NM, KF, and ST contributed to writing sections of the manuscript.

### Conflict of interest statement

The authors declare that the research was conducted in the absence of any commercial or financial relationships that could be construed as a potential conflict of interest.
