# Supplementary material for: Further Characterization of the Capsule-Like Complex (CLC) Produced by Francisella tularensis Subspecies tularensis: Protective Efficacy and Similarity to Outer Membrane Vesicles
Source: Front Cell Infect Microbiol. 2018 Jun 15;8:182. doi: 10.3389/fcimb.2018.00182 (PMC6013578; doi:10.3389/fcimb.2018.00182)
Supplement: Supplementary file 1 [file Table_1.DOCX]

**Supplementary Table 1. Chaotropic buffers used for extraction of the CLC**

**Extraction buffer Modification**

1X PBS pH 7

10 mM ethylenediaminetetraacetic acid (EDTA)

10 mM ethylene glycol tetraacetic acid (EGTA)

100 mM HEPES pH 2, 4, 6, 7.5, 8 and 12

100 mM Tris buffer pH 7.2

200 mM glycine-HCl pH 2, 3, 4

0.5% β-mercaptoethanol in 10 mM NaCl

10 mM CaCl_2_

100 mM HEPES +10 minute, 65°C incubation

1 M urea

1 M guanidine-HCl

10 mM NaCl
