# Supplementary material for: Further Characterization of the Capsule-Like Complex (CLC) Produced by Francisella tularensis Subspecies tularensis: Protective Efficacy and Similarity to Outer Membrane Vesicles
Source: Front Cell Infect Microbiol. 2018 Jun 15;8:182. doi: 10.3389/fcimb.2018.00182 (PMC6013578; doi:10.3389/fcimb.2018.00182)
Supplement: Supplementary file 2 [file Table_2.DOCX]

**Supplementary Table 2. Amino acid composition of CLC extract from LVS.**

| **Amino Acid Type** | **Percent** |
| --- | --- |
| Acidic^a^ | 29.0 |
| Hydrophobic^b^ | 53.0 |
| Basic | 8.7 |
| Uncharged Polar | 9.3 |

__________________________________________________________________

^a^The acidic amino acids consisted of aspartic and glutamic acids.

^b^The predominant hydrophobic amino acids were glycine, proline, valine, isoleucine, norleucine, and leucine.
