# Supplementary figures and images for: Further Characterization of the Capsule-Like Complex (CLC) Produced by Francisella tularensis Subspecies tularensis: Protective Efficacy and Similarity to Outer Membrane Vesicles
Source: Front Cell Infect Microbiol. 2018 Jun 15;8:182. doi: 10.3389/fcimb.2018.00182 (PMC6013578; doi:10.3389/fcimb.2018.00182)

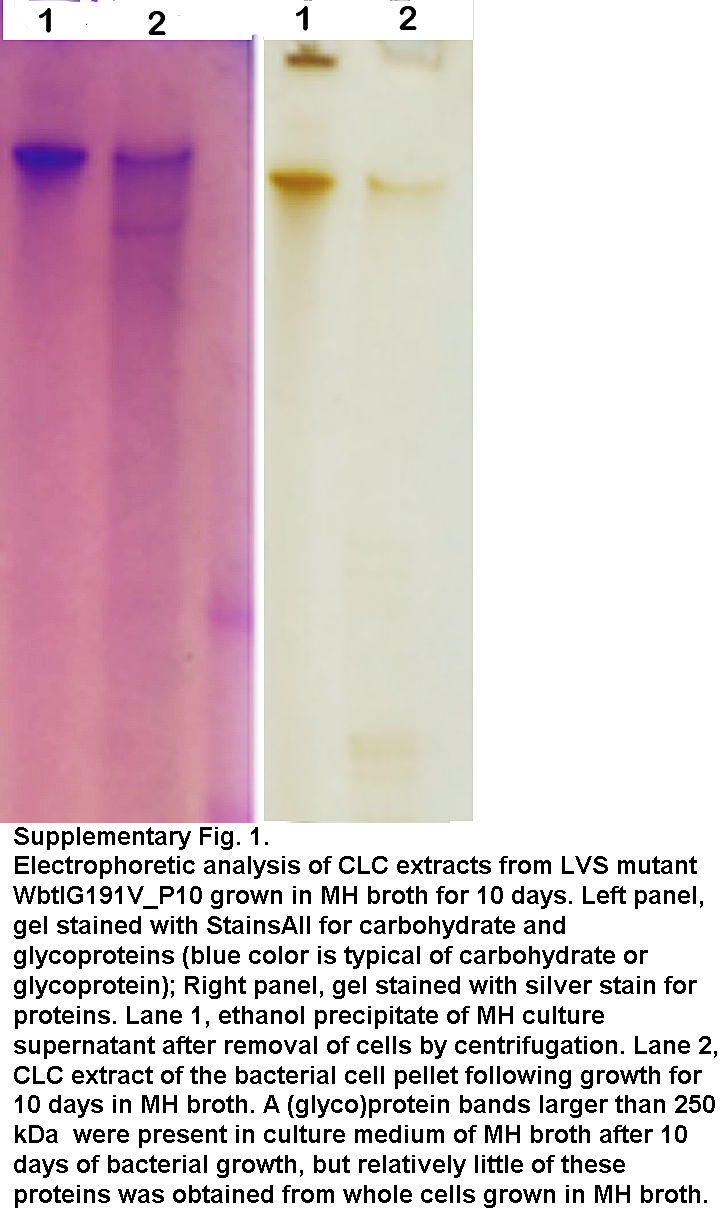

Supplement: Supplementary file 3 [file Image_1.tiff]
